# Supplementary material for: Deciphering cell states and genealogies of human haematopoiesis
Source: Nature. 2024 Jan 22;627(8003):389–98. doi: 10.1038/s41586-024-07066-z (PMC10937407; doi:10.1038/s41586-024-07066-z)
Supplement: Supplementary file 1 — This file contains Supplementary Figs. 1–11, Table 1, Methods and Notes. [file 41586_2024_7066_MOESM1_ESM.pdf]

nature portfolio

https://doi.org/10.1038/s41586-024-07066-z

Supplementary information

Deciphering cell states and genealogies of human haematopoiesis

In the format provided by the
authors and unedited

Table of Contents

Table of Contents *1*

Supplementary Figures *3*

Supplementary Fig. 1: ReDeeM protocol optimization and quality control. 4

Supplementary Fig. 2: Additional analysis of agreement of closeness 5

Supplementary Fig. 3: Additional batch of CRISPR-based tracing experiment. 6

Supplementary Fig. 4: mtDNA mutations that support phylogenetic and lineage analysis. 7

Supplementary Fig. 5: Validation of fine-scale human hematopoiesis lineage tracing 8

Supplementary Fig. 6: Single-cell multiomics TF motif analysis for HSC subpopulations 10

Supplementary Fig. 7: Validation of network propagation-based clonal output analysis. 11

Supplementary Fig. 8: Schematic of eUMI collision false negatives. 12

Supplementary Fig. 9: Histogram of the number of shared mutations between nearest neighbors for cells in Young-1 dataset. 13

Supplementary Fig. 10: Simulation of the robustness of cell-cell distance inferred by mtDNA mutation 14

Supplementary Fig. 11: mtDNA mutation variation and frequency in young-1 dataset. 15

Supplementary Fig. 12: Gating strategy for Young-1: FACS enrichment for live BMMC, HPC, and HSC cells. 17

Supplementary Tables *18*

Supplementary Table 1 18

Supplementary Methods *19*

ReDeeM Method 19

Cell fixation, permeabilization and tagmentation 19

Single-cell joint RNA and ATAC library preparation 19

mtDNA enrichment and library preparation 20

Sequencing for ReDeeM libraries 20

Single-cell eUMI-based mtDNA mutation discovery 20

Experimental condition optimization with cell hashing 22

Species mix experiment 23

CHIP mutation detection 23

Single-cell multi-omics clustering 23

Integrative analysis of CRISPR-based lineage tracing and ReDeeM 24

HSC clonal behavioral trajectory ... 26
Gene Set Enrichment and TF Motif enrichment ... 26
Supplementary Notes ... 28
Comparative analysis of ReDeeM, mtscATAC and MAESTER ... 28
Model mutation collision rate ... 30
Validation of mtDNA mutations for lineage tracing using single colony WGS ... 30
Comparison of mtDNA mutation detectability between single colony WGS and ReDeeM ... 31
Mitochondrial dynamics and mutation dropout analysis ... 32
Assessment of functional impact from mtDNA mutations ... 33
Enumeration of multifaceted ReDeeM validations ... 34
Supplementary references ... 36

Supplementary Figures

Supplementary Fig. 1

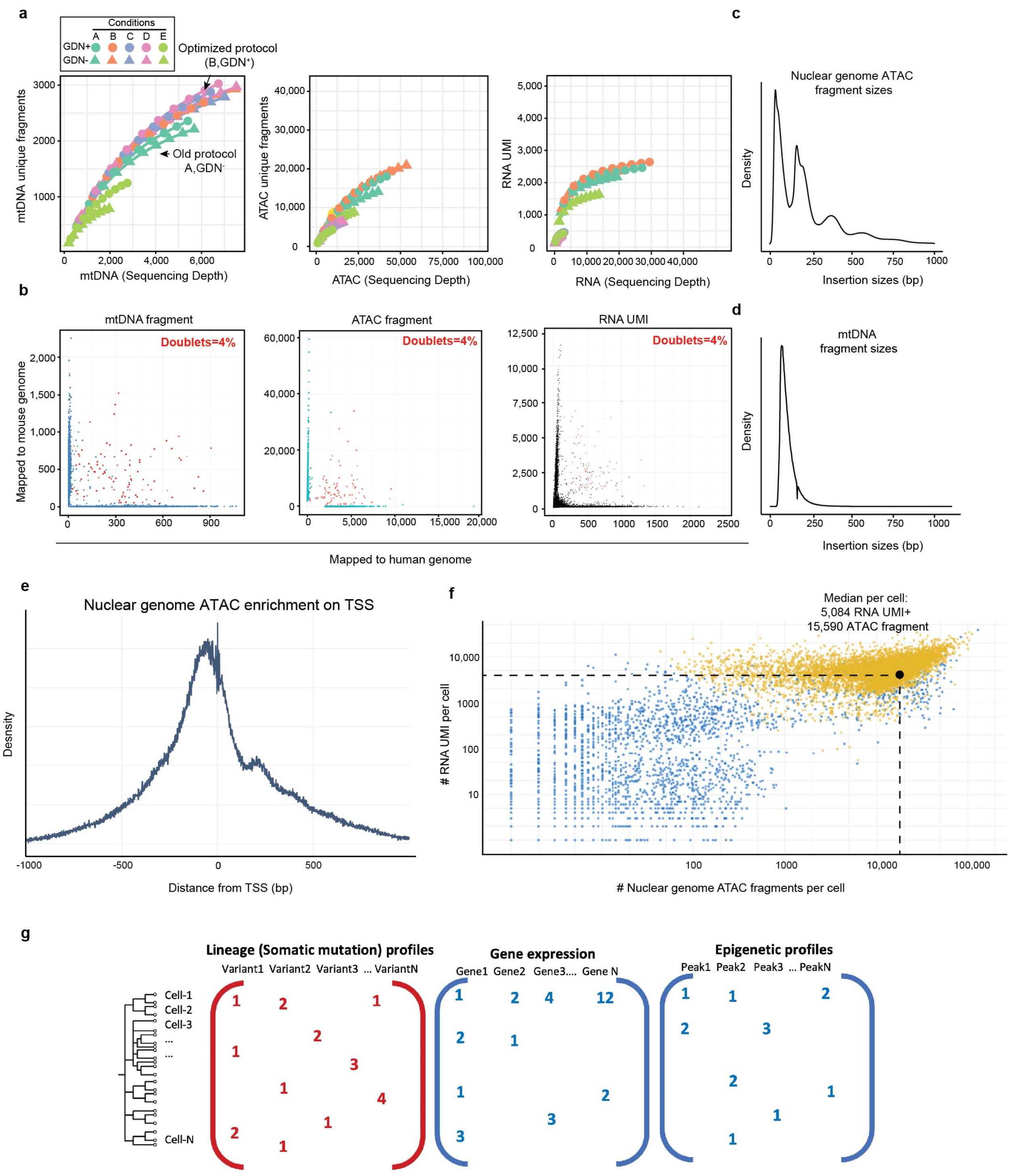

Supplementary Fig. 1: ReDeeM protocol optimization and quality control.

a, The recovery efficiency rate of mtDNA, ATAC-seq, and RNA-seq across 10 different experimental conditions in fixation, permeabilization, and tagmentation was determined to identify the optimal condition. A (0.1% FA,10min RT,0.1% NP40 5 min, 1% BSA) B (0.1% FA,10min RT,0.1% NP40 5 min, 0.01% BSA); C (1% PFA,30 min RT,0.1% NP40 5 min, 1% BSA); D (1% PFA,30 min RT,0.1% NP40 5 min, 0.01% BSA); E (1% PFA,30 min RT, No lysis, 0.01% BSA). All conditions are tested with and without 1% glyco-diosgenin (GDN) in tagmentation. **b,** Human-mouse species mix experiment. 4% of the droplets were doublets in all three modalities. **c-d**, Tn5 insertion size distribution in **c,** nuclear genome DNA or **d,** mitochondrial genomes. **e,** Aggregated ATAC-seq fragments near transcription start sites (TSS). **f,** Distribution of joint-profiled single cells (dots) based on the number of ATAC nuclear fragments versus the number of RNA transcripts UMI per cell using ReDeeM. **g,** Schematic of the ReDeeM data structure.

Supplementary Fig. 2

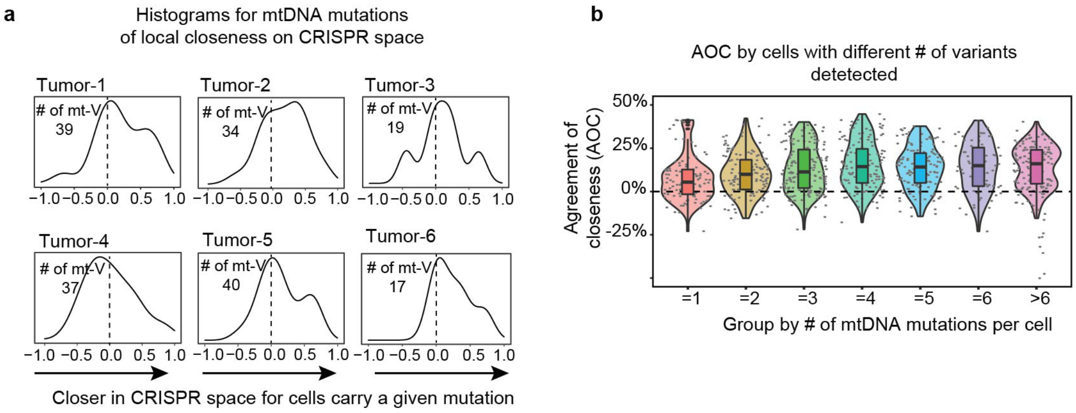

Supplementary Fig. 2: Additional analysis of agreement of closeness

a, Histogram of mtDNA mutations of the local enrichment score (x axis) in CRISPR indel-based distance matrix (T1-T6, Related to Extended Data Fig. 5c) . **b**, The distribution of "agreement of closeness" is grouped by cells with different mtDNA mutation detected. The more mtDNA mutations detected per cell, the better agreement with CRISPR indel-based method. n=904 cells. Boxplot displays data from the 25th to 75th percentile, and whiskers extending to the minimum and maximum within 1.5 IQR.

Supplementary Fig. 3

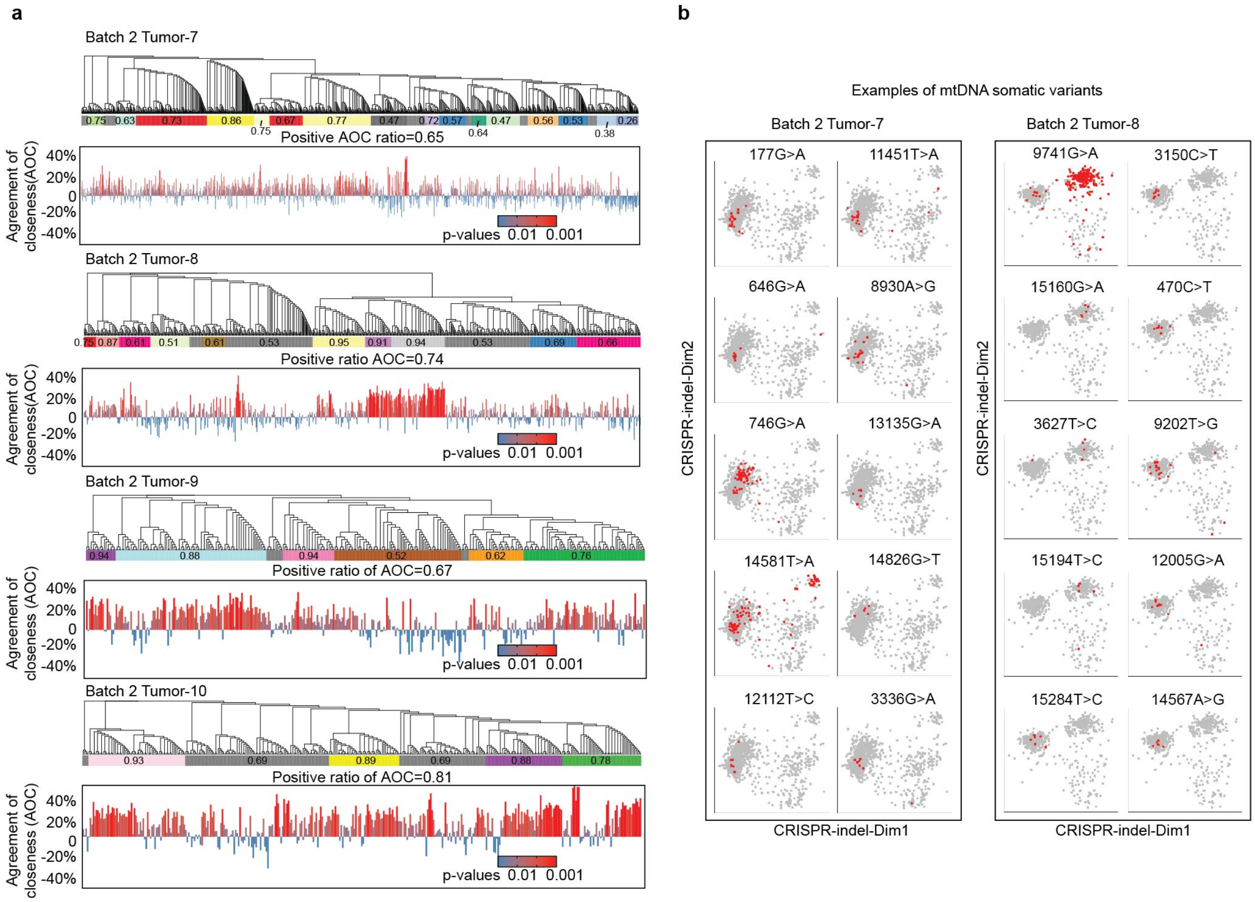

Supplementary Fig. 3: Additional batch of CRISPR-based tracing experiment.

a, The analysis in Extended Data Fig. 5e is reproduced for an additional batch with 4 tumors (T7-T10), showcasing the phylogenetic trees (clonally grouped) along with the agreement of closeness. **b**, The same analysis as Extended Data Fig. 5c is presented, demonstrating 20 examples of mtDNA mutations for T7 and T8.

Supplementary Fig. 4

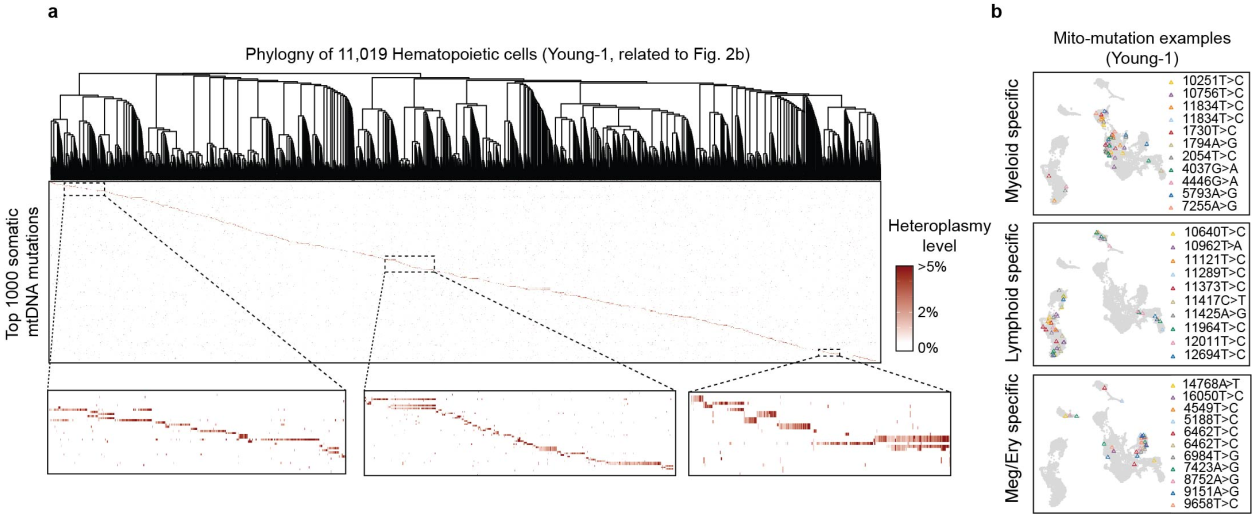

Supplementary Fig. 4: mtDNA mutations that support phylogenetic and lineage analysis.

a, Top 1000 informative mtDNA mutations supporting the phylogenetic tree. Zoom-in views are shown below. Many of the fine-scale phylogenetic structures are supported by more than one mtDNA mutation. **b**, Examples of mtDNA mutations with restricted lineage preference are highlighted on cell-state UMAP.

Supplementary Fig. 5

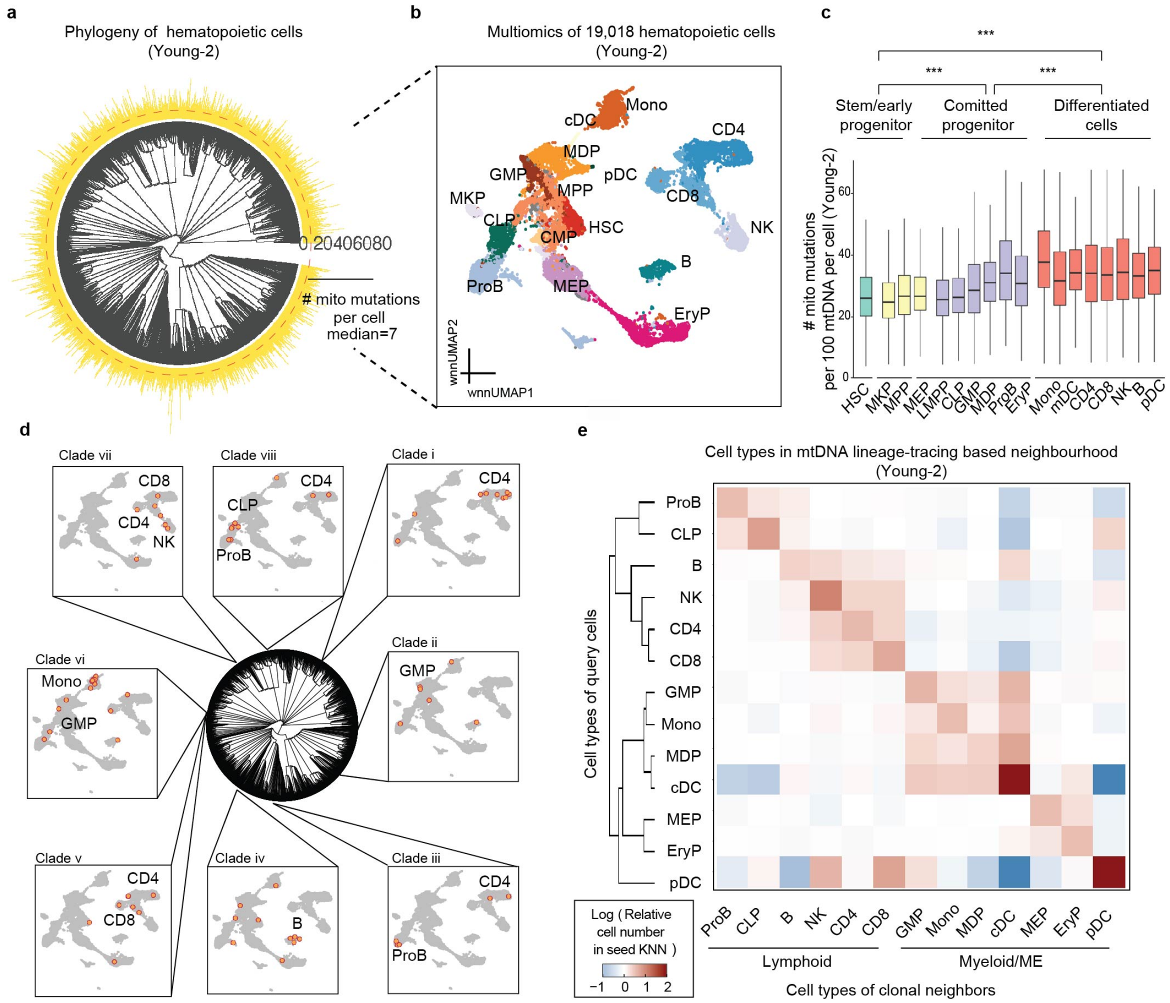

Supplementary Fig. 5: Validation of fine-scale human hematopoiesis lineage tracing

Same analyses of Fig. 2 were reproduced on donor young-2. **a,** Phylogenetic tree for hematopoietic cells of young-2. The number of sharable mtDNA mutations for each cell is indicated. **b,** Joint multi-omics clustering of young-2. Weighted-nearest neighbour UMAP (wnnUMAP) shows combined ATAC and RNA profile for each cell. **c,** Measurement of mtDNA mutation burdens across different cell types in young-2. n=19,018 cells. Boxplot displays data from the 25th to 75th percentile, and whiskers extending to the minimum and maximum within 1.5 IQR. *** indicates p-value<$2.2*10^{-16}$, derived from two-sided Wilcoxon rank sum test**.** **d**, Integrative analysis between phylogenetic tree and multi-omics-based cell types in young-2. Examples of cell type restricted local clades are shown (clade i to clade viii). **e,** same as main Fig 2g for donor young-2. Analysis of cell type origins based on lineage informative mtDNA mutations. Color intensity indicates the proportion of each target cell type (horizontal axis) within the mtDNA mutation-based k nearest neighbourhood (KNN) of the query cell type (vertical axis).

Supplementary Fig. 6

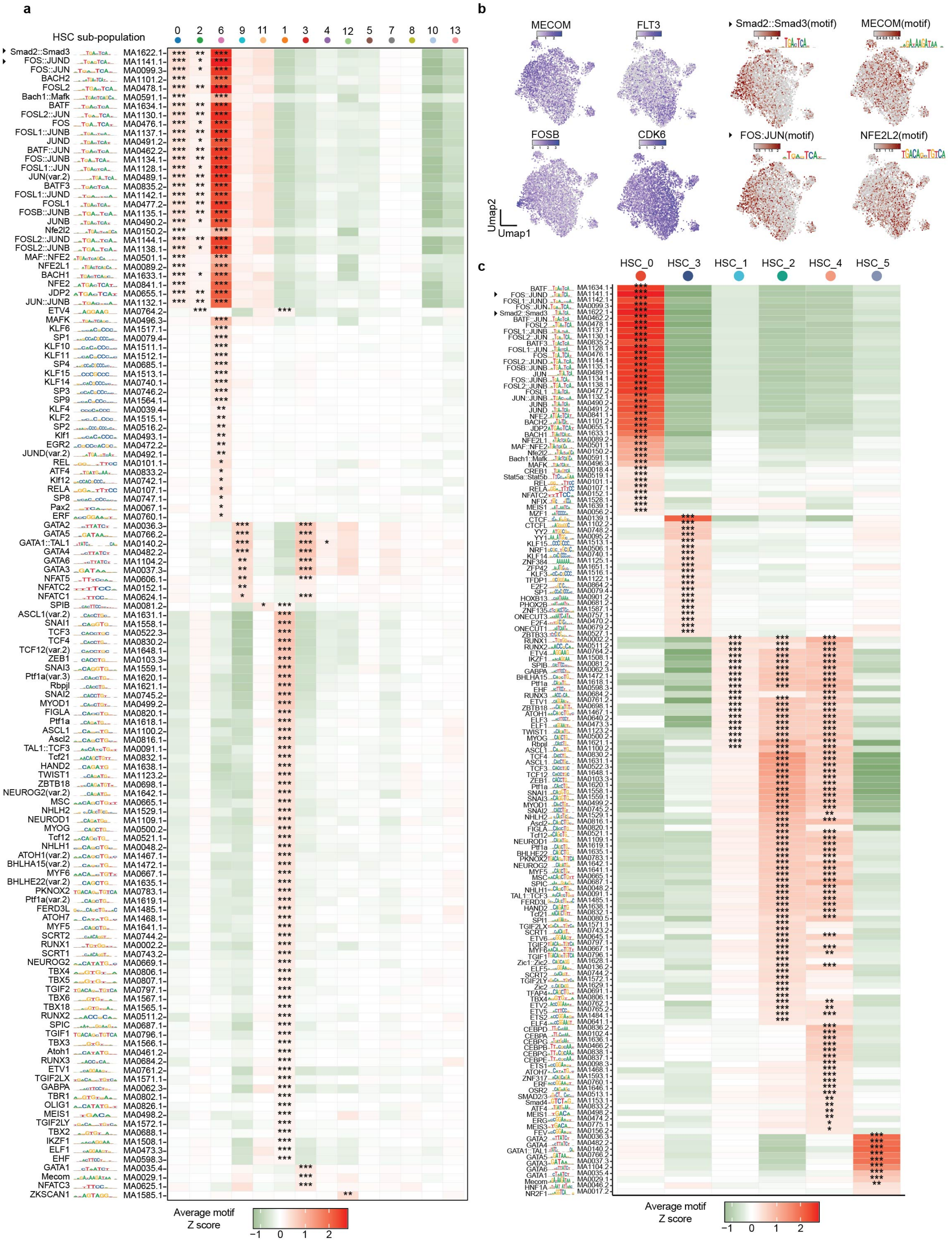

Supplementary Fig. 6: Single-cell multiomics TF motif analysis for HSC subpopulations

Related to Extended Data Fig. 7. **a-b** for analysis of donor young-1, **c** for analysis of donor young-2**. a,** Top examples of HSC subpopulation-specific transcription factor DNA binding motifs, based on ATAC modality for donor young-1. Triangles indicate the ones discussed in the manuscript. **b,** Examples of differentially expressed genes(blue) and differential transcription factor binding motifs (brown) across HSC subpopulations on the wnnUMAP for donor young-1.**c**, same analysis as a, for donor young-2

Supplementary Fig. 7

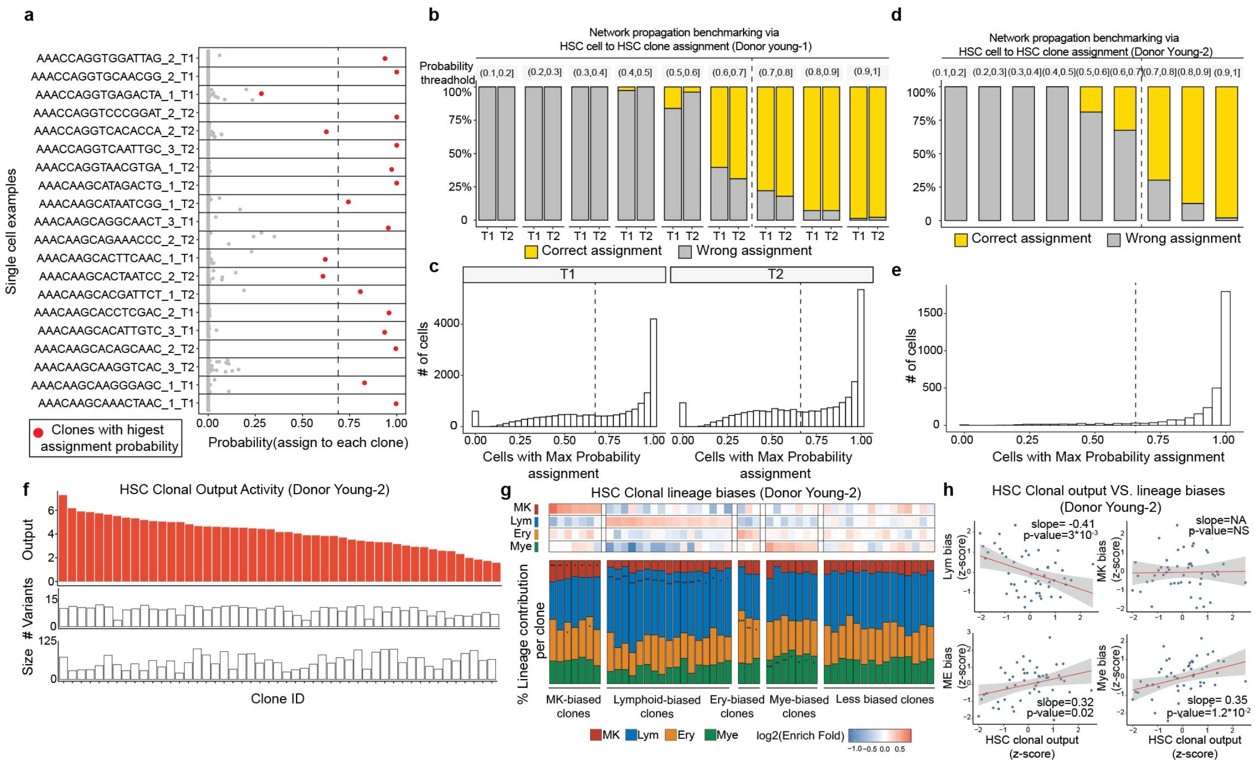

Supplementary Fig. 7: Validation of network propagation-based clonal output analysis.

Related to main Fig. 4. **a,** The assignment probability of progeny cell examples (20 random cells) to all 78 HSC clones. The probability is determined from network propagation. The HSC clones with highest probability are colored in red, but only the ones above the threshold 0.7 are assigned for downstream analysis. **b,** Benchmarking the assignment accuracy in donor young-1. The predicted clonal assignments for HSCs using network propagation are compared to the "ground truth", the actual HSC clones. The accuracy is computed for different probability thresholds. **c**, The histogram that summarises the number of cells with different maximum assignment probability in donor young-1. **d-e,** Reproduce the same analysis of b and c for donor young-2. **f-h,** Reproduce the analysis of Fig. 4b,e,f in donor young-2. **f**, Summary of HSC clonal output activity in young-2. **g**, For each HSC clonal group, the percentage of the progenies that differentiate into one of four main lineages: megakaryocyte (MK), lymphoid,Mega-Erythroid (ME), and myeloid (Mye) cells. **h,** Correlation between HSC clonal output activity and clonal lineage biases in young-2. Error bands 95% confidence level interval for predictions from a linear model. P-values are derived from Wald-test.

Supplementary Fig. 8

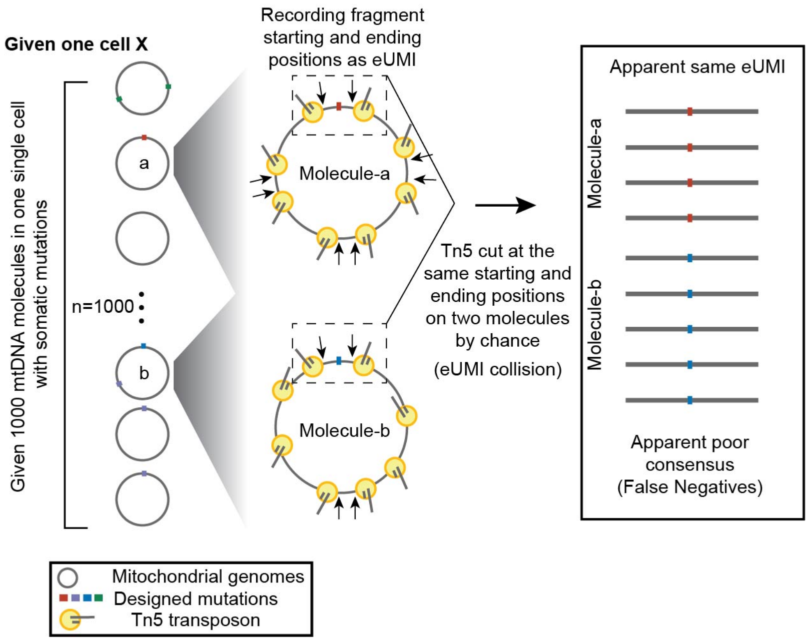

Supplementary Fig. 8: Schematic of eUMI collision false negatives.

When two different molecules are incorrectly labelled as one, the consensus mutation calling tends to be poor due to the apparent inconsistency, and thus it leads to false negatives, or the dropout of a true mutation.

Supplementary Fig. 9

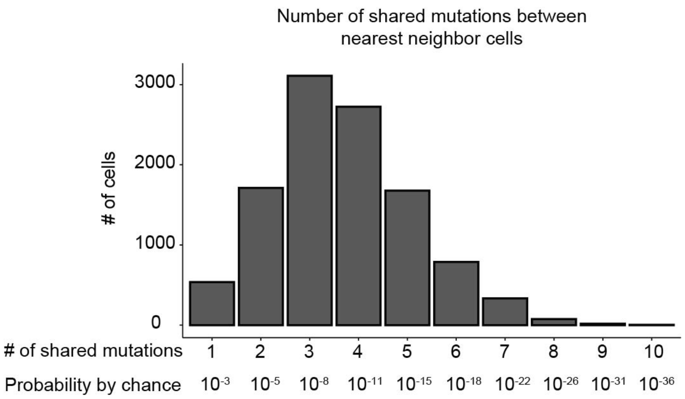

Supplementary Fig. 9: Histogram of the number of shared mutations between nearest neighbors for cells in Young-1 dataset.

The probability of sharing # mutations between two cells by chance is calculated by randomizing 10 mutations across the 16,569 bp mitochondrial genome per cell.

Supplementary Fig. 10

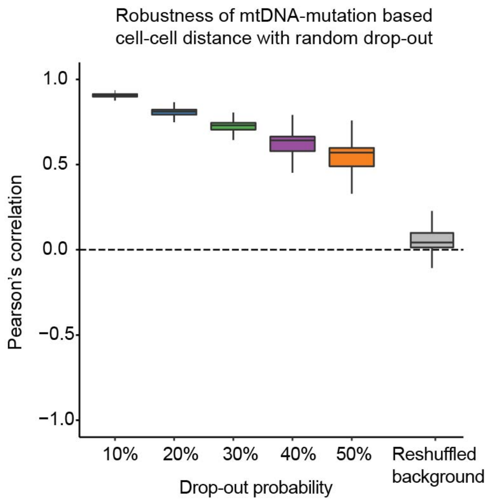

Supplementary Fig. 10: Simulation of the robustness of cell-cell distance inferred by mtDNA mutation

mtDNA mutations have been partially dropped with various probabilities modelled by binomial distribution. The Pearson's correlation coefficient of cell-cell distances between independent drop-out simulations are shown. The randomly reshuffled mutation matrix is used as background. n=9519 cells for each box. Boxplot displays data from the 25th to 75th percentile, and whiskers extending to the minimum and maximum within 1.5 IQR.

Supplementary Fig. 11

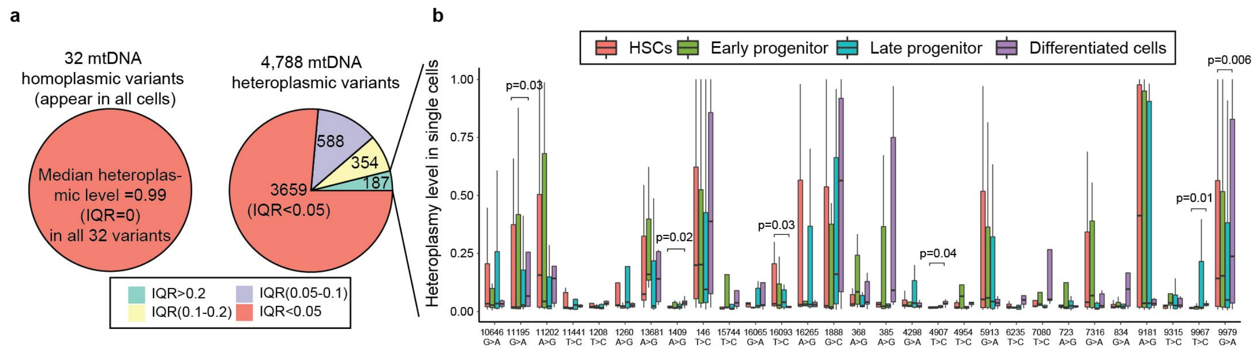

Supplementary Fig. 11: mtDNA mutation variation and frequency in young-1 dataset.

(**a**) Summary of mutations with different levels of variation in heteroplasmy. IQR: Interquartile Range. (**b**) Heteroplasmy level change during differentiation for mtDNA mutation with high IQR. n=16,008 cells. Boxplot displays data from the 25th to 75th percentile, and whiskers extending to the minimum and maximum within 1.5 IQR. P-values are derived from Wilcoxon rank sum test.

Supplementary Fig 12

a

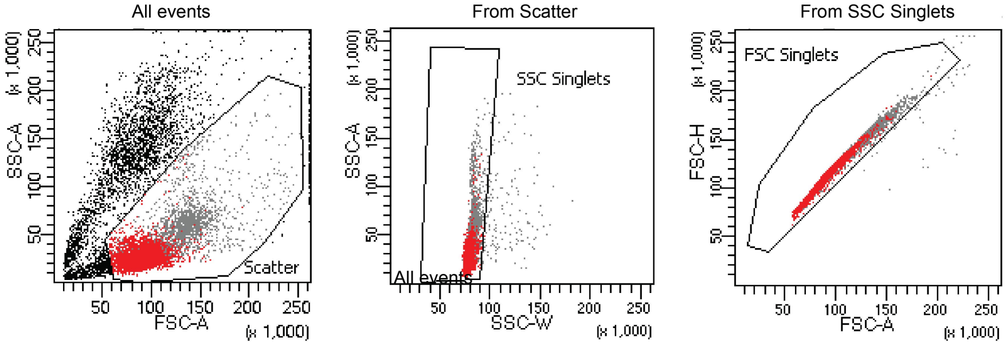

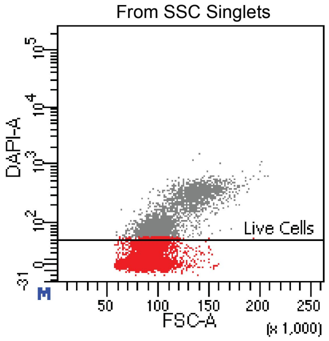

Tube: BMMC Unstained

Population	#Events	%Parent	%Total
All Events	11,965	####	100.0
Scatter	8,621	72.1	72.1
SSC Singlets	8,463	98.2	70.7
FSC Singlets	8,411	99.4	70.3
Live Cells	4,631	55.1	38.7

b

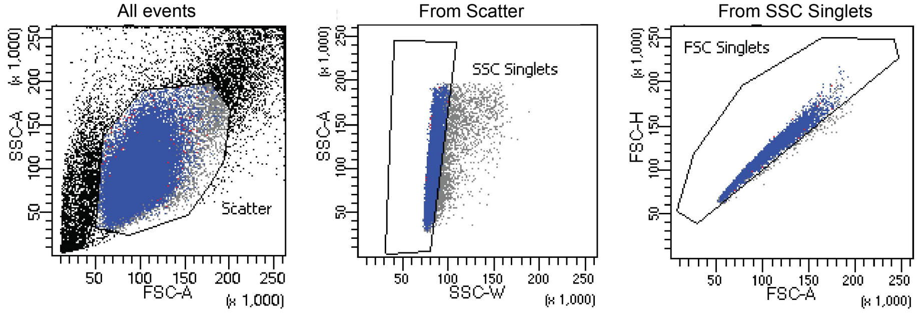

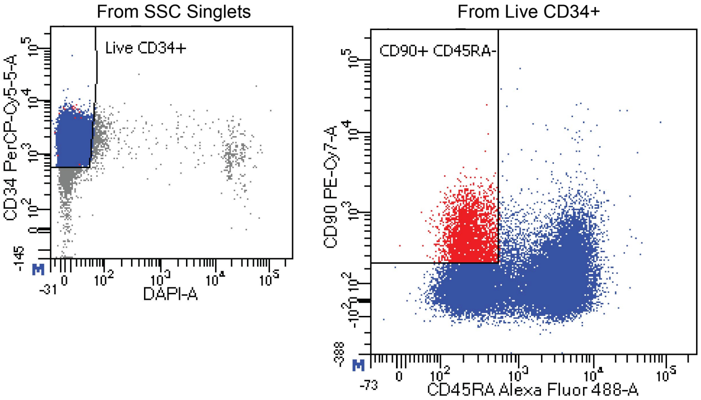

Tube: HSPC

Population	#Events	%Parent	%Total
All Events	47,468	####	100.0
Scatter	36,571	77.0	77.0
SSC Singlets	34,371	94.0	72.4
FSC Singlets	34,218	99.6	72.1
Live CD34+	31,816	93.0	67.0
CD90+ CD45RA-	3,323	10.4	7.0
NOT(CD90+ CD45RA-)	28,493	89.6	60.0

Supplementary Fig. 12: Gating strategy for Young-1: FACS enrichment for live BMMC, HPC, and HSC cells.

(**a**) Gating and sorting strategy for live BMMC(DAPI-) (**b**) Gating and sorting strategy for live HSC (CD34+CD45RA-CD90+) and HPC (CD34+ NOT CD45RA-CD90+)

Supplementary Tables

Supplementary Table 1

Donor ID	Batch	Age	Sex	Samples profiled	sample	CHIP mutation	Cell #
young-1	Main	31	F	BMMC, $CD34^+$, $CD34^+CD45RA^-CD90^+$	Twice (4 Mon)	NA	54,221
young-2	Main	26	M	BMMC, $CD34^+$, $CD34^+CD45RA^-CD90^+$	Once	NA	34,721
aged-1	Main	76	M	BMMC, $CD34^+$	Once	Not detected	20,496
aged-2	Main	78	M	BMMC, $CD34^+$	Once	DNMT3A-R736H(11%)	25,717
young-3	Addition	22	F	BMMC, $CD34^+$	Once	NA	1,725
young-4	Addition	21	M	BMMC, $CD34^+$	Once	NA	1,132
young-5	Addition	29	F	BMMC, $CD34^+$	Once	NA	1,462
young-6	Addition	20	F	BMMC, $CD34^+$	Once	NA	2,046
young-7	Addition	32	F	BMMC, $CD34^+$	Once	NA	2,444
aged-3	Addition	81	M	BMMC, $CD34^+$	Once	NA	1,777
aged-4	Addition	85	F	BMMC, $CD34^+$	Once	NA	983
aged-5	Addition	69	M	BMMC, $CD34^+$	Once	ASXL1-Q373X (28.3%)	1,042

Note: BMMCs indicate extracted bone marrow mononuclear cells, $CD34^+$ cells are enriched cells for hematopoietic stem and progenitor cells (HSPCs), $CD34^+CD45RA^-CD90^+$ are enriched cells for hematopoietic stem cells (HSCs, see Methods for more details). The main batch is the 4 donors (young-1, young-2, aged-1, aged2) that are mainly analyzed and presented in the manuscript. The Additional batch is processed in a pooled manner for validation.

Supplementary Methods

ReDeeM Method

Cell fixation, permeabilization and tagmentation

The fixation and permeabilization conditions are similar to our previous reports with some modifications. We tested 10 different conditions which are detailed in later sections. The final recipe is as follows. For each sample, cells were resuspended in 0.1% formaldehyde in DPBS, with 0.04% bovine serum albumin (BSA) and 0.2 U/µl RNase Inhibitor (RI), and were incubated at room temperature for 10 minutes. The fixation was then quenched by 0.125M glycine, at room temperature for 3 minutes. Cells were centrifuged 500g for 5 min at 4C and washed once by 3ml cold PBS/RI (DPBS with 0.04% BSA, 0.2U/ul RI). Cells were then incubated for 5 minutes on ice in 1ml permeabilization buffer (10mM Tris, 10mM NaCl, 3mM MgCl, 0.1% NP40, 1mM DTT, 1 U/µl RI), which were followed by adding 3 ml cold Wash buffer (10mM Tris, 10mM NaCl, 3mM MgCl,0.02% BSA, 1mM DTT, 0.2 U/ul RI). Cells were then centrifuged and resuspended in 1X Nuclei Buffer (10x Genomics, Catalog # PN2000207). Cells were counted and diluted to 2,800 cells/µl, which served as the input for tagmentation reaction. A modified tagmentation protocol was applied as follows, a volume of 5 µl of cell suspension from previous step was mixed with 5.5 µl ATAC buffer B(10x Genomics, Catalog # PN2000193),1.5 µl ATAC-GDN(ATAC buffer B with 1% glyco-diosgenin (or GDN, Avanti Polar Lipids, 850525P-500MG)), and 3 µl Tn5 (ATAC Enzyme B, 10x Genomics, Catalog # PN2000265). The reaction was in 15ul and incubated at 37°C for 1 hour and immediately processed to the next step.

Single-cell joint RNA and ATAC library preparation

After tagmentation, cells were immediately processed for GEM generation & barcoding following 10x Genomics Chromium Next GEM Single Cell Multiome ATAC + Gene Expression User Manual (version CG000338 Rev A, 10x Genomics) with Step 2. Next, the Post GEM Incubation Cleanup and Pre-Amplification PCR were performed following the same User Manual with Step 3 and Step 4. The pre-amplified library was eluted in 100ul EB buffer instead of 160ul.

For RNA library prep, we took an aliquot of 35 ul pre-amplified library and followed the same User Manual (version CG000338 Rev A, 10x Genomics) from Step 6 and Step 7.

We developed an in-house protocol to generate the massive ATAC library with enough material for both direct sequencing and mtDNA hybridization enrichment. For each sample, 8 reactions of the following were performed (each in 50 ul). 5 µl pre-amplified product, 25 µl NEBnext mix (Catalog M0541L), 2.4 µl 10 µM Nextera N70X primer (**Supplementary Data 1**), 2.4 µl 10X SI PCR primer B (Catalog #PN2000128), 0.5 µl 100X SYBR Green (Catalog #S7563), and 14.7 µl water. The thermocycler program started with 98°C 45 s., followed by the 6-12 cycles of: 98°C for 10 s., 63°C for 30 s., and 72°C for 20 s.; The actual cycle numbers are determined using the amplification curve and the qPCR was stopped when the curve started to reach plateau. The

qPCR products (50ul * 8 wells) were combined and purified using SPRI beads (Catalog #B23318) with size selection 0.4X~1.55X. This is the ATAC library products. The expected yield is 2,000 ng to 4,000 ng.

mtDNA enrichment and library preparation

We designed 4 sets of DNA probes for mtDNA hybridization enrichment, with each probe set covering the whole mitochondrial genome and containing 138 biotinylated oligos that are 120 bp long. The 4 sets of probes ((v1, v2, v3, and v4) are staggered by 30 bp with each other to ensure good coverage on junction areas (**Supplementary Data 1**). The hybridization started with 500 ng ATAC library products from the last step for each probe set. In total, 4 independent reactions were performed in parallel and combined at the end (2,000ng input in total). The detailed incubation and wash methods followed the Tube Protocol in IDT xGen Hybridization Capture of DNA Libraries protocol. Briefly, 500 ng input was combined with Cot DNA and purified by SPRI beads, which was eluted by hybridization mix that contains hybridization buffer, hybridization buffer enhancer, one of the probe set, as well as xGen™ Universal Blockers NXT (Catalog #1079584). The hybridization reaction was performed as below: 95C 30 seconds followed by 65C for 16 hours. The biotin-streptavidin Dynabeads purification was carried out on Day 2 using xGen Hybridization and Wash Kit. The Dynabeads after capturing and washing were used for final quick qPCR amplification (3-5 cycles). The reaction mix is as below: 20ul beads, 25ul KAPA HiFi HotStart ReadyMix (Catalog #KK2601), Primer 2.4ul P5 (**Supplementary Data 1**), Primer P7 (**Supplementary Data 1**), 0.5ul 100X SYBR Green (Catalog #S7563). The PCR program started with 98°C 45 s, followed by the 3-5 cycles of: 98°C for 10 s., 63°C for 30 s., and 72°C for 20 s. The final enriched mtDNA was purified by SPRI beads (1.6X)

Sequencing for ReDeeM libraries

For each sample, all three libraries were sequenced on Illumina NovaSeq S4 300 cycle kit. The sequencing length was as below. Read1: 149nt, i7: 8nt, i5: 24nt, Read2: 149nt. We expected to sequence 20,000~30,000 raw reads per cell for RNA libraries and ATAC libraries, while 50,000~60,000 raw reads for enriched mtDNA libraries.

Single-cell eUMI-based mtDNA mutation discovery

Unique molecule identifier (UMI) labels each original DNA molecule and has been widely used for rare variant calling to minimize artefacts that arise during PCR amplification and sequencing. The addition of UMI has been demonstrated to substantially improve the sensitivity and accuracy of mutation discovery (from 10^{-3} up to 10^{-7}), and therefore it has been widely used in studying rare somatic mutations[69].

Natural somatic mutation in mtDNA appear more frequently than in the nuclear genome but the allele frequencies of most lineage informative mutations are still considered to be rare. We reasoned that the ability to accurately detect those low-frequency mtDNA mutations would be critical for lineage tracing technology that reconstructs fine-scale phylogenetic relationships.

We found that the single-cell droplet based mtDNA fragments already had sufficient information to distinguish original single molecules, i.e., we are able to use UMI without introducing extra UMI barcodes. To be specific, the paired-end sequencing for each fragment provides information of single-cell barcode (CellBC) as well as Tn5 cutting sites on the left (start) and on the right (end). We term the CellBC-start-end as endogenous UMI (eUMI). We reasoned that the eUMI is sufficient to label unique original mtDNA molecules. Given a single cell (by CellBC), the Tn5 enzyme cutting sites that create fragments during transposition reaction are most likely unique across different mtDNA molecules. The theoretic collision rate to use eUMI is in function of the mtDNA number per cell and the Tn5 cutting efficiency. We tested *in silico* whether the probability of cutting exactly the same starting and ending position in two different mtDNA molecules in the same cell, namely eUMI collision, is low enough to make such assumptions to use the eUMI to label a single molecule. We randomly simulated 10 times the Tn5 fragmentation given a cell with variable mtDNA copies. The cutting sites were randomly selected on the mtDNA genome based on real Tn5 cutting preference. The parameters used in these simulations were *N* (The number of mtDNA copies per cell), S (Size of mtDNA, 16.9kb), and E (Tn5 cutting efficiency, or the dropout rate). We let N vary between 10 to 50,000 molecules per cell, while we set E as 0.1, based on empirical data. The result of the simulation showed that if the mtDNA is around or lower than a couple of thousand per cell, the eUMI is a robust method to label unique molecules with limited collision. Notably, based on prior knowledge, the mtDNA copy numbers in hematopoietic cells are safely in that range[82]. Specifically, when there are around 1000 mtDNA moleculaes per cell, the collision rate is 3%, which means 3% of reads are incorrectly labelled as the same molecule but in fact should be different molecules. We consider this collision rate is low enough because the impact is negligible. As illustrated in **Supplementary Fig. 8**, when two different molecules are incorrectly labelled as one, the consensus mutation calling tends to be fail due to the apparent inconsistency, and thus it leads to false negatives, or the dropout of a true mutation. 3% of mutation dropout due to eUMI collision will have negligible effects to the downstream analysis, which are modelled and discussed further below (**Supplementary Notes, Supplementary Fig. 10**).

The mutation calling pipeline is by following steps: 1, Data Preprocessing and eUMI grouping. 2, Consensus filtering. 3, Strand bias removal. 4, Lineage informative variants identification (**Extend Data Fig. 1i**). First, we masked the nuclear mitochondrial DNA, or NUMT regions on the auto chromosomes as previously reported followed by mapping using bowtie2[28]. We only used the reads that were uniquely mapped to the mitochondrial genome (samtools view -bf 2 -q30). We filtered for bases by sequencing quality with Q30 or higher. The bases that were sequenced in both read1 and read2 were processed separately from the bases with only one direction sequenced. After the preprocessing steps, we then extracted the CellBC-start-end (eUMI) for each sequenced fragment and generated the eUMI groups for the consensus variant calling. The following consensus filtering parameters were applied. The minimum eUMI group size was 2 or 3, depending on whether the given base was read by one direction or both directions (90% of bases are covered by both R1 and R2 using ReDeeM protocol, **Extended Data Fig. 1c**). The consensus score must be 100% if eUMI group size was smaller than 4, whereas the consensus score threshold is 75% if the eUMI group size is equal or larger than 4. After the consensus filtering, we used a binomial modeling method to evaluate and remove the significantly strand-

biased mutations (p value <0.01, fold>2). The NUMT is a potential confounder for the real mtDNA mutations. Previously in bulk studies, heterozygous NUMT mutation was modeled to control this contamination[83]. Here, following the same principle, we examined the mutation allele numbers per cell. If a mutation appeared to have 2 or more than 2 alleles in one single cell, that mutation was considered as mtDNA mutations. Otherwise, the mutation would be removed. Finally, we removed homoplasmic mutations that appeared in all single cells as well as all the heteroplasmic mutations that were only detected in one single cell, both of which were not lineage informative. All the parameters used above can be adjusted to control the stringency in ReDeeM-V and ReDeeM-R.

Experimental condition optimization with cell hashing

As mentioned above, to achieve the best mtDNA capture efficiency while maintaining the data quality of gene expression and chromatin accessibility, we designed a pooled experiment to simultaneously test different conditions of fixation, permeabilization, and tagmentation. The readouts include 1) number of unique mtDNA fragments per cell, 2) number of gene counts per cell. 3) number of unique accessible chromatin fragments per cell. 4) Enrichment of accessible fragments on transcription starting sites (TSS)

To minimize the batch effects and maximize the cost effectiveness of the test experiments, we split the same pool of cells (BMMCs) into 8 aliquots for 8 experimental conditions which were uniquely cell hashed using Biolegend TotalSeq™ Hashtag reagents. The hashing procedure was as follows. First, 5 million cells were washed once by DPBS and centrifuged at 500g for 5 min. The cell pellet was then resuspended in 400 μl Hash-staining buffer (2% BSA and 0.01% Tween in PBS) and split into 8 tubes coated with Hash-staining buffer. For each sample aliquot, 5μl of Human TruStain FcX™ (BioLegend catalog# 422302) was added followed by incubation at 4°C for 10 min for blocking. Then, to each sample aliquot, 1 μl of the uniquely barcoded CellHash antibody was added (TotalSeq™-A0257 to -A0265), specific to each sample aliquot, along with 50 μl of staining buffer (consisting of 2% FBS in PBS). The sample aliquots were incubated at 4°C for 30 minutes. Subsequently, 3 ml of Hash-staining buffer was added to each tube, followed by centrifugation at 4°C for 5 minutes at 500g. After removing the supernatant, the washing step was repeated twice.

The hashed cells were processed with different conditions before GEM generation and barcoding. Specifically, the following conditions were tested. First, we reasoned that mild lipid detergent during tagmentation may increase the accessibility of Tn5 enzyme into mitochondria. Accordingly, we set two groups, one with and the other without adding 1% GDN (glyco-diosgenin, Avanti Polar Lipids 850525P-500MG) in tagmentation. In each group, we tested different fixation conditions including 0.1% FA, 0.2% FA, 1% PFA, methanol, etc. We also tested with high or low levels of BSA concentration (The detailed condition is shown in **Supplementary Fig. 1**). After tagmention, all cells were combined equally and loaded into one 10X genomics lane. Next, 10X Multiome standard protocol was followed as mentioned above, except adding 0.2uM Hash-Spikin primer (**Supplementary Data 1**). All three ReDeeM libraries were generated and sequenced.

The cell hashing data was resolved using pymulti from scEASYMode (https://github.com/johnnyUCSF/scEasyMode) . The comparative analysis was performed for each modality across all testing conditions by controlling the same sequencing depth (**Supplementary Fig. 1**).

Species mix experiment

With the modified protocol, we tested whether it may affect the leakage of mtDNA and RNA, since we fixed and permeabilized the cells. To examine that, we performed a species mixing experiment by 1:1 mixing a human K562 cell line and mouse lung cancer cells. The mixed cells were processed with the standard ReDeeM protocol and the leakiness and doublet rates were measured on all three modalities (**Supplementary Fig. 1**).

CHIP mutation detection

Amplicon sequencing (Archer VariantPlex) was performed for bulk detection of Clonal Hematopoiesis of Indeterminate Potential (CHIP) mutations. Amplicons covered 40 different genes and spanned mutation hotspots containing sites of known driver mutations in myeloid malignancies. DNA was purified from the bone marrow samples, and this DNA was directly probed using the Archer amplicon sequencing. In brief, purified DNA was first subjected to enzymatic fragmentation using 10nM of DNA in 50µl of 10mmM Tris-HCL pH 8.0. End repair was then performed on the entire sample volume and purified with AMPure XP beads (Beckman Coulter, #A63881) using 2.5X volume for a total of 125µl of AMPure XP beads per reaction and eluted into 20µl of 10mmM Tris-HCl pH 8.0. 20µl of eluted solution was then used for incorporation of unique molecular barcodes. These barcodes were performed in two ligation steps. The first ligation step was performed at 37°C for 15min and cleaned with 2.5X AMPure beads and eluted into 42µl 10mM Tris-HCl pH 8.0. 40µl of elution was used for incorporation of P5 Illumina sample index, and reactions were resuspended in 50µl ArcherDX Ligation Cleanup Beads (SA0210) and 50µl of ArcherDX Ligation Cleanup Buffer (SA0209). Pelleted samples were washed with 200µl ArcherDX Ligation Cleanup Buffer (SA0209) and eluted into molecular biology grade deionized water. Following amplification, Illumina P7 indexes were incorporated, and samples were purified using 0.8X AMPure XP beads and eluted into 20µl of 10mM Tris-HCl pH 8.0.

Final eluted samples were then quantified using KAPA ROX Low Library Quantification DNA Control Standard (Roche Material Number: 7960336001). Libraries were then pooled with 20% PhiX and sequenced on an Illumina NextSeq 500/550 using a Mid-Output Kit v2.5 with 150 cycles.

Single-cell multi-omics clustering

The data pre-processing for the joint single-cell RNA and ATAC data was performed using 10X Genomics data pre-processing software Cell-ranger-arc. The basic quality control analysis was as follows: RNA UMI: 1,000~25,000 transcripts per cell; unique ATAC fragment: 1,000 ~ 70,000; fragment on peak minimum percentage: 10%; minimum mtDNA copies per position per cell: 10; Finally, the possible doublets are removed by Amulet (using default parameter)[84].

Unsupervised community detection-based clustering was performed on weighted nearest neighbour (WNN) using Seurat[85]. Cell types are firstly annotated by gene expression using the latest signature (van Galen under review) which was further validated by sorted bulk RNA-seq data (https://sankaranlab.shinyapps.io/geneExpression/). The ATAC unique fragments for each clustered cell type were further aggregated to generate pseudo-bulk ATAC tracks using in-house scripts. The single cell level motif analysis was performed using ChromVar using the JASPAR human transcription factor (TF) database[77,86]. The deviation of the TF frequency was computed for each cell for the downstream analysis, including the visualization in **Fig 2d**.

Integrative analysis of CRISPR-based lineage tracing and ReDeeM

We previously created and reported a Kras;Trp53(KP)-drive lung adenocarcinoma lineage-tracer mouse model[36]. We used this model and designed a dual lineage tracing experiment where we detected both engineered CRISPR-based evolving barcodes on the nuclear genome as well as the naturally occurring mitochondrial somatic mutations by ReDeeM in the same single cells (**Extended Data Fig. 5a**). The dual tracer experiment would generate single-cell data with 5 modalities in the same cell (Cell Hashing, scRNA, scATAC, mtDNA, Target Site). The scRNA and scATAC modalities were used to identify cells that passed the quality control. The Cell Hashing data were used to split the data into individual tumors (6 tumors in batch-1 and 4 tumors in batch-2) using an in-house python script. The Target Site data were indels created by Crispr-based lineage tracer and would be used to compare with the mtDNA mutation-based lineage inferences.

Standard ReDeeM pipeline was used for pre-processing mtDNA data as described above. The Target Site data was pre-processed using Python package Cassiopeia[79]. Briefly, reads with identical cellBC and UMI were collapsed into a single, error-corrected consensus sequence representing a single-expressed transcript. Consensus sequences were identified within a cell based on a maximum of 10 high-quality mismatches (PHRED score greater than 30) and an edit distance less than 2 (default pipeline parameters). UMIs within a cell reporting more than one consensus sequence were resolved by selecting the consensus sequence with more reads. Each consensus sequence was aligned to the wild-type reference Target Site sequence using a local alignment strategy, and the intBC and indel alleles were called from the alignment. Cells with fewer than 2 reads per UMI on average or fewer than 10 UMIs overall were filtered out. These data were summarized in a molecule table which records the cellBC, UMI, intBC, indel allele, read depth, and other relevant information. Then, character matrices were formed summarizing indel information across the N cells in a population and their M cut-sites. Characters (i.e., cut-sites) with more than 80% missing information or containing a mutation that was reported in greater than 98% of cells were filtered out for downstream analysis.

The character matrices were then used to compute a hamming distance (hamming distance in Cassiopeia) that describes the cellular lineage relationships. Next, we examined whether the cells carrying the same given mtDNA somatic mutation tend to be neighbors in CRISPR indel-based hamming distance matrix. For any mtDNA mutation present in more than 3 cells, we computed the average cell-cell distance in CRISPR-based distance matrix for these carrier cells. We also

computed the average distance for the same number of cells that are randomly picked as the expected distance. The mutation “local enrichment score” is calculated by subtracting the rank of the observed average distance from the expected rank. This difference is then normalized by the expectation, resulting in a value between -1 and 1 (**Supplementary Fig. 2a**), where a positive score suggests that the observed distance for cells with the same given mutation is shorter than random reshuffle. For visualization of individual mtDNA mutation examples, the CRISPR-based hamming distance was then dimension reduced using multidimensional scaling (MDS) using function *cmdscale* in R. The mtDNA mutations were overlaid on the MDS map (**Extended Data Fig. 5b-c, Supplementary Fig. 3b**).

To systematically benchmark the cellular lineage distance between mtDNA mutation-based and the CRISPR indel-based recorder, we generated two independent k nearest neighbourhoods based on mtDNA mutation-derived Jaccard distance (Graph $\mathbf{G^{mt}}$) and CRISPR indel-derived hamming distance (Graph $\mathbf{G^{cr}}$). We devised a metric “Agreement of closeness” or AOC to quantify the degree to which the mtDNA mutation-based lineage relationships were supported by CRISPR tracer (**Extended Data Fig. 5e**). Given a single cell X, we firstly identified k (default is 15) nearest neighbours M_1,M_2.. M_k in mtDNA mutation derived graph $\mathbf{G^{mt}}$. Then we computed the average distance from M_1,M_2..M_k to the cell X on CRISPR derived graph $\mathbf{G^{cr}}$. The ranks of these distances on $\mathbf{G^{cr}}$ (The closest is indicated as rank #1) were compared with that from randomly picked k cells. The random process was repeated 1000 times and the rank % closer toward cell X in observed data than expected was defined as “agreement of closeness” or AOC. (i.e. Positive value of AOC indicates a closer distance to cell X than expected, while negative score indicates farther). For example, if the real rank of nearest neighbor from $\mathbf{G^{mt}}$ in $\mathbf{G^{cr}}$ is 8th while the random reshuffled rank is 16th, the AOC is (16-8)/32= 25%. The empirical p value was also generated by i permutations (default is 1000). For any given cell X.

$$Rank_{observed} = rank(\frac{1}{20}\sum_{k=1}^{20} distance(M_k \in G^{cr}, X \in G^{cr}))$$

$$Rank_i = rank(\frac{1}{20}\sum_{k=1}^{20} distance(random\ cell\ k \in G^{cr}, X \in G^{cr}))$$

$$Agreement\ of\ closeness = \frac{1}{1000}\sum_{i=1}^{1000} \frac{(Rank_i - Rank_{observed})}{Total\ cell\ number}$$

Next, we built the phylogenetic tree based on the mtDNA mutation data using neighbor joining algorithm. The trees were visualized along with the AOC metrics which provide single-cell level assessment of agreement (**Extended Data Fig. 5e, Supplementary Fig. 3a**).

We also provided assessment of agreement at clonal level using the following two methods:

First, we identified the clonal groups using the same method as described for HSC clonal analysis (via *Add_tree_cut function* in ReDeeM-R). We defined the "positive AOC ratio" as the fraction of cells within a clonal group that exhibited positive agreement between ReDeeM and CRISPR-

based inference (AOC > 0). This metric was calculated for each clonal group as well as the entire trees for each tumor.

Second, we provided the Adjusted Rand Index (ARI) to examine the consistency of the clonal clustering/grouping. To minimize the biases due to clustering resolutions, we computed the ARI for multiple clonal resolutions (using Louvain clustering resolution 0.2~1.2) for CRISPR indel-based and mtDNA mutation-based distance. The Adjusted Rand Indices were calculated using the adj.rand.index function in the fossil package.

HSC clonal behavioral trajectory

The analysis of HSC clonal output and lineage biases above will generate 5 “clonal behavior” metrics for each HSC clone: output activity; myeloid biases; erythroid biases; megakaryocyte biases; lymphoid biases. We rank all HSC clones according to each of the “clonal behavior” scores to generate 5 HSC clonal behavioral trajectories respectively. Along each trajectory, we identify differentially expressed genes or peaks based on poisson regression as follows:

$$GeneExpression/PeakIntensity = e\hat{}(\beta 0 + X * \beta + \beta 6 * log(TotalUMI))$$

- *GeneExpression/PeakIntensity*: Represents the dependent variable, which is the gene expression level or the ATAC peak intensity.
- *β0*: The intercept term, representing the baseline gene expression/ peak intensity level.
- *X*: A vector containing the scores of interests, including Output Activity Score (O), Myeloid Biases Score (M), Ery Biases Score (E), MK Biases Score (K), and Lym Biases Score (L).
- *β*: A vector containing the coefficients corresponding to each score in X, representing the effects of the behavioral scores by gene expression/peak intensity.
- *β6*: The coefficient representing the effect of log(TotalUMI) on gene expression.
- *log(TotalUMI)*: The logarithm of TotalUMI, a measure related to the total number of UMI of gene expression (or total fragment number of ATAC) in the analysis.

We applied Wald test to determine the p-value of each coefficient, and the FDR is computed by the q-value package in R. The differential genes/peaks are identified by the threshold of FDR<0.01. The differential peaks are clustered using hierarchical clustering based on the peak signal changes along with the clonal behavioral scores (slope).

Gene Set Enrichment and TF Motif enrichment

The nearby genes for all differential peaks are identified by Homer (annotatePeaks.pl). The gene function enrichment analysis was performed by integrating MSigDB[87,88]. All functional term lists were read into R using the package ‘gage’. For any given group of genes, a binomial test (binom.test) was performed iteratively through all annotated functional terms. P-values for enrichment were further adjusted using the qvalue package. Enrichment terms were ranked by q values. The most representative top terms were selected and visualized using heatmaps. On each heatmap, q values of enriched terms were visualized by colour intensity as enrichment scores (**Extended data Fig. 8d**).

The DNA binding motifs of differential peaks were analysed by FIMO scanning with the JASPAR human TF motif database followed by a binomial test across the 5 peak categories (**Extended Data Fig.8e**).

Supplementary Notes

Comparative analysis of ReDeeM, mtscATAC and MAESTER

The standard mtscATAC-seq calls mitochondrial mutations from the ATAC library using 10X Genomics ATAC kit without generating a specific mtDNA library. The ReDeeM protocol generates ATAC, RNA and mtDNA libraries using 10X Genomics multiome kit. The major improvement of ReDeeM was the mtDNA hybridization capture enrichment and the use of eUMI for consensus variant calling. To perform a comparison with minimized batch/kit effect, we started with the same pre-amplified library derived from the same sample (young-1 HSPC) using multiome kit. We prepared the ATAC library and processed the data with mgatk package to approximate the mtscATAC results (and a similar protocol on multiome called DOGMA-seq[29]), whereas we enriched the mtDNA library and processed the data through the ReDeeM-R/V packages to represent the standard ReDeeM results. The ReDeeM and the approximate mtscATAC results were compared for the mappability, mtDNA genome coverage, mutation calling sensitivity, etc (**Extended Data Fig. 3**).

Of note, the coverage measure in ReDeeM is different from our previously published metric[28,29]. In mtscATAC, we counted the unique reads number of paired-end sequencing twice, counting Read1 and Read2 separately, whereas ReDeeM is a single-molecule based method, counting reads with the same eUMI only once as one molecule and only considering high quality reads that exceed Q30 score. For example, in this manuscript 50x indicates there are 50 unique molecules covering one position in one cell. This method of counting is more stringent, and 50X in ReDeeM would be approximately 100X if the previous counting method was used.

To compare the ReDeeM with MAESTER[31], we also started with the same pre-amplified library derived from a single sample (young-2 BMMC) using multiome. The preamplified library was used to generate both the mtDNA library for ReDeeM-R/V analysis as well as the cDNA library from which the MAESTER library was produced. The MAESTER library was then sequenced and analyzed with maegatk as previously described[31]. The ReDeeM and MAESTER results were compared for mtDNA genome coverage, mutation calling sensitivity, mutational signature, etc (**Extended Data Fig. 3**). Mutational signature was analyzed by the function *MutationProfile.bulk* in ReDeeM R.

Below summarizes the comparisons across ReDeeM, MAESTER, and mtscATAC from several aspects in principle (**Extended Data Fig. 3a**)

- ReDeeM and mtscATAC detect variants from mtDNA, while MAESTER uses mtRNA. In general, mtDNA and mtRNA have consistent variant representation. But the coverage from mtDNA is more even, and there are no artifacts due to RNA editing in mtDNA. Therefore, mtDNA is a more reliable source in finding true variants. On the other hand, methods using mtRNA can be more broadly applied, since single-cell RNA-seq kits are more available and commonly used.

- The use of UMI or eUMI can substantially increase the mutation calling sensitivity (**Extended Data Fig. 2, simulation**). Both MAESTER and ReDeeM applied UMI/eUMI in mutation calling, whereas mtscATAC does not apply UMI.
- Of note, ReDeeM uses eUMI to label a unique mtDNA molecule, while the MAESTER uses UMI to label a unique mtRNA molecule. In principle, labeling unique mtDNA molecules is considered to be less biased and more quantitative compared to mtRNA labeling. This distinction arises from the variability in transcription levels that can affect mtRNA. For instance, certain mitochondrial genomes may exhibit lower transcription activity, or their transcripts may be less stable compared to other mitochondrial genomes within the same cell. This variability becomes particularly relevant when a mutation exerts functional effects on transcription processes.
- Cost-effective deep sequencing is critical for mutation calling. Both ReDeeM and MAESTER generate a specific mitochondrial library for sequencing (near 100% reads are mitochondrial reads in the sequencing library). But mtscATAC does not. Typically, in the mtscATAC library there are only ~10-15% reads covering the mitochondrial genome. Therefore, mtscATAC need 10 fold more reads to achieve the same sequencing depth on mitochondria genomes.
- The coupling with other -omic profiling is valuable. mtscATAC is ATAC only; MAESTER is RNA only; ReDeeM is coupled with both ATAC and RNA in the same cell. They are all useful, depending on the research goals.

We compared the ReDeeM and the approximate mtscATAC protocol using the young-1 CD34+ sample. We generated the "mtscATAC" data from the same donor using non-enriched ATAC library (from the same multi-ome assay for a fair comparison). As discussed above, the "mtscATAC" library only contains 10% reads that are mapped to the mitochondrial genome (**Extended Data Fig 3b**). The median coverage is 14.3X in "mtscATAC" library and 51.7X in ReDeeM library (defined as # of unique mtDNA fragments that cover each position per cell). Using mgatk for mtscATAC data, we identified 311 mutations (**Extended Data Fig 3d)**. In contrast, ReDeeM identified 4,831 confident mutations that are further validated by expected mutational signatures (**Fig 1**).

We compared the ReDeeM and MASTER protocol using the young-2 BMMC sample. We generated the "MAESTER" library using the cDNA from the same multi-ome assay for a fair comparison. As is shown in the Extended Data Fig 3e, the reads distribution on the MAESTER library is biased in some regions. The average coverage in MAESTER is lower than ReDeeM. We took the top 307 mutations identified by MAESTER and the 4,087 mutations by ReDeeM. Both showed expected mutational signatures (**Extended Data Fig. 3g**). Although only a small fraction of the mutations identified by ReDeeM is well covered in MAESTER, they show great consistent (78% of the well covered mutations are supported by ReDeeM, **Extended Data Fig. 3h**).

Taken together, ReDeeM has several technical and conceptual advances compared to previously described methods. Nonetheless, depending on the research goal, all these approaches are valuable and in some cases are complementary.

Model mutation collision rate

Mutation collision could potentially occur when identical mutations spontaneously manifest in two unrelated cells purely by coincidence. We have modeled the probability of "mutation collision" by random simulation. From ReDeeM data, we typically detect around 10 mutations in each single cell across the mitochondrial genome 16,569 bp. By random simulation, the probability that two cells have 1 shared mutation by chance is 0.6%. Notably, ReDeeM uses the combination of multiple mutations with a median of 4 mutations that are shared with their nearest neighbors. The probability of sharing 4 mutations by coincidence would be very low, 1.4*10-11 (**Supplementary Fig. 9**).

Validation of mtDNA mutations for lineage tracing using single colony WGS

We re-analyzed 42 single colony WGS data from an MPN patient with known JAK2 V617F somatic mutation, which serve as an ideal case to validate both clonal and subclonal consistency (**Extended Data Fig. 6a**)[37]. This dataset is deeply sequenced, and the mitochondrial genome is well covered (**Extended Data Fig. 6b-c**). We used the nuclear genome somatic mutations to infer a phylogenetic tree as previously described and compared mitochondrial mutations against the tree. Notably, it is challenging to accurately detect mitochondrial mutations in WGS data because most genuine mitochondrial mutations are of low frequency, which makes them hard to differentiate from PCR/sequencing errors (**Extended Data Fig. 2a**). We grouped candidate mitochondrial mutations based on variant allele frequencies and assessed whether the group of mutations are trustable based on the mutational signature compared to the expected enrichment pattern in C > T and T > C transitions[35]. As anticipated, the lower the variant allele frequency, the weaker the mutational signature pattern is (**Extended Data Fig. 6e**). We pinpointed the top 5% of mutations (comprising 41 mutations) that display clean enrichment patterns, classifying them as confident mitochondrial mutations. After excluding homoplasmic mutations (N=36, present in all colonies) and singular mutations (N=2, found in only one colony), three confident heteroplasmic somatic mutations remain. Strikingly, all these three confident mutations align almost perfectly with the lineage tree inferred from the nuclear genome (**Extended Data Fig. 6f-g**). Specifically, 15562A>G and 14581T>C clearly mark the expanded JAK2 mutant clade, while notably 5237G>A is further restricted to a subclade of the JAK2 expansion, providing subclonal information. The combination of these mutations together provides robust lineage information. Indeed, ReDeeM utilized multiple mutation combinations and thus can be resilient to a certain level of mutation dropout (**Supplementary Fig. 10**). Furthermore, we examined the less confident (less stringent) mitochondrial mutations, although the signal is much noisier, they successfully revealed the closer distance (i.e., more shared mutations) within the expanded JAK2 mutant clone (**Extended Data Fig. 6h**). Together, despite the limited ability to detect mitochondrial mutations from WGS, there is evident consistency with the nuclear-based tree at both the clonal and subclonal levels.

Our observations are broadly consistent with a recent report[38] from Chapman et al. that re-analyzed mtDNA mutations from single hematopoietic colony WGS. Consistent with our analyses of the Van Egeren et al. data, the Chapman et al. data suggest that, with this method, low VAF

mtDNA variants are contaminated by mutations with mutational signatures that diverge from those characteristic of mitochondria suggestive of sequencing artifacts or mutations acquired in vitro, while mtDNA mutations with higher VAF display the canonical mitochondrial mutational signature and show significant concordance with the phylogenetic trees derived from nuclear somatic mutations. Therefore, the use of mtDNA mutations detected at high VAF from single colony WGS data for lineage tracing are consistent between our analysis and that of Chapman et al. However, it is challenging to investigate rarer mtDNA mutations in single colonies. A more sensitive WGS method (such as UMI based WGS) will be needed to achieve sufficient detectability. Of note, ReDeeM provides substantially enhanced sensitivity for rare mtDNA mutations and we have demonstrated its capability of fine-scale lineage tracing through multiple lines of evidence.

Comparison of mtDNA mutation detectability between single colony WGS and ReDeeM

It is worth noting that mitochondrial mutations and nuclear genome mutations from single colony WGS is not directly comparable due to inherent limitations in detectability. Detecting true somatic mutations from the nuclear genome is considerably more straightforward than detecting those in the mitochondrial genome. If a heterozygous somatic mutation is localized within the nuclear genome of a given cell, the single colony starts with a variant allele frequency (VAF) of 50%. In the post-expansion WGS data, once germline mutations are excluded, the search primarily targets heterozygous mutations with a VAF of 50% (**Extended Data Fig. 2a** top panel). This methodology is both reliable and efficient. In contrast, mutations in the mitochondrial genomes, which have hundreds to thousands of copies in the single cell, often begin with a markedly low initial variant frequency, perhaps around 1% or even lower. Consequently, despite the cellular expansion into a single colony, the mitochondrial mutations most likely preserve this low frequency (**Extended Data Fig. 2a** bottom panel). In our simulations (more details below), even without factoring in PCR errors, these low-frequency mitochondrial mutations cannot be easily distinguished from sequencing errors (**Extended Data Fig. 2c,d**). This explains the observed high background noise, or weak mutational signature of mitochondrial mutations derived from WGS, especially as the variant allele frequency gets lower (**Extended Data Fig. 6e**). As a comparison, ReDeeM, due to the incorporation of eUMI, efficiently corrects errors at single molecule level **(Extended Data Fig. 2c,e)**. We examined all 3,808 mitochondrial mutations identified by ReDeeM from one donor HSPC dataset. All these mutations, regardless of variant allele frequency, show very clean mutational signatures enriched in C>T and T>C transitions, as expected (**Extended Data Fig. 6i-j**). Together, ReDeeM is able to detect true mitochondrial mutations with enhanced sensitivity and accuracy, including the low frequency ones, compared to single colony WGS. In future, while challenging, it would be of interest to explore new methods that incorporate UMI into WGS in low-input single colonies with a specific focus on achieving ultrasensitive detection of mitochondrial mutations.

For the simulation analysis of mtDNA mutations in WGS, one single cell with 1000 mtDNA copies was simulated. We designed 50 rare mutations with different variant allele frequencies (0.1%~0.5%). We randomly placed these mutations into the simulated mtDNA genomes. The simulated mtDNA (with mutations) were then in silico fragmented, amplified (assuming no PCR

errors) and sequenced by in-house script and ART sequencing simulator. The resulting fastq files were then processed by eUMI-based ReDeeM or the conventional method without using eUMI. The ReDeeM parameters used here were consistent with the main analysis. Using conventional method (or "single colony WGS" in **Extended Data Fig. 2**), the mapped reads were compared to the reference. The variant allele frequencies were computed for all candidate mutations (i.e. both real mutations and errors). The real mutations were highlighted. As expected, most low heteroplasmy mtDNA mutations cannot be distinguished from error background using conventional methods but clearly stand out after the ReDeeM-V pipeline (**Extended Data Fig. 2d,e**).

Last, conceptually, single colony assay operates on the premise that no alterations take place during ex vivo growth and that this growth is consistently balanced. It is suitable for nuclear genome WGS but comparing it with the mitochondrial genome may not be equitable because mitochondrial genome can accumulate a greater number of subclonal somatic mutations, owing to its higher mutation rate, even during ex vivo growth to some extent[27]. This suggests that mtDNA mutation data could offer additional unique insights into subclonal (ex vivo) lineages, compared to nuclear genome mutations.

Mitochondrial dynamics and mutation dropout analysis

To achieve robust lineage tracing, ReDeeM employs binarizing mitochondrial mutation counts, a data transformation step ensuring that lineage distances between cells are determined solely by the number of shared mutations, without being affected by the fluctuations in heteroplasmy levels (see above for more details about matrix C_{bin}). This approach helps mitigate potential biases stemming from drift due to mitochondrial dynamics.

While our method has reduced the direct impact of heteroplasmy level fluctuations, the drift in heteroplasmy level could lead to mutation detection dropout, i.e., false negatives, an issue that cannot be readily addressed by binarization. In principle, ReDeeM has the capability to withstand a certain degree of dropout. This is because ReDeeM uses combinations of mutations to infer lineage connections rather than on a singular mutation, benefiting from the enhanced mutation detectability. Theoretically, multiple mutations can compensate one another during partial dropout that can minimize the impact of false negatives. To validate this, we conducted a mutation dropout simulation. Essentially, we proposed that there's a chance (ranging from 10% to 50%) for a cell to lose a mutant allele copy for each mutation due to drift or detection challenges. By repeatedly simulating mutation dropouts, we measured the correlation of cell-to-cell lineage distances inferred by ReDeeM between different independent simulated dropout datasets. As anticipated, even with a 50% dropout rate, the correlation remained strong (with a median Pearson's correlation of 0.57, **Supplementary Fig. 10**). This suggests that ReDeeM can accommodate a certain extent of mutation dropout, ensuring robust lineage inference.

Furthermore, we systematically investigated the degree of variation in mtDNA mutation heteroplasmy levels across cells before the binarization, using young-1 data as an example (**Supplementary Fig. 11**). The 32 "homoplasmic variants" are present in all cells and show no

variation with a median heteroplasmy level of 0.99 (or 99%), and Interquartile range (IQR)=0. Of the 4,788 "heteroplasmic variants", which are present in a subset of cells, the majority exhibited low heteroplasmy level and minimal heteroplasmy variation (4,601 mutations had an IQR<0.2, with a median heteroplasmy level of 0.02). We further analyzed the 187 mutations with an IQR>0.2 by examining their heteroplasmy level variations across HSCs, early progenitors, late progenitors, and differentiated cells, focusing on mutations present in at least 3 cells in each category. Most of these mutations did not display significant changes in heteroplasmy levels across the differentiation stages. These analyses suggest that the majority of mtDNA mutations have limited fluctuations of heteroplasmy level across cells. For a few that do exhibit variations, we have not seen functional selections that influence cell fate decisions during differentiation in normal hematopoiesis.

Assessment of functional impact from mtDNA mutations

To benchmark the potential functional impact from mtDNA mutations, we used the young-1 dataset as an example (given that this dataset has the largest representation of cell types). We first analyzed the mtDNA mutation distributions across the mitochondrial genome and observed a fairly uniform distribution spanning different genes and non-coding regions. We annotated all mitochondrial coding genes and the D-loop region which contains regulatory elements for mtDNA replication and transcription. No significant mutation enrichment or depletion were observed based on specific genome regions (**Extended Data Fig. 4a**).

Next, we classified all mutations into 4 categories: missense, nonsense, synonymous, and non-coding. All these mutation types were observed (**Extended Data Fig. 4a**). To measure the potential functional selections on mitochondrial mutations, we computed the dN/dS ratios (via dndscv package in R[89]), a normalized ratio of nonsynonymous to synonymous mutations (a score of 1.0 represents overall neutrality; a ratio <1.0 suggests an excess of negative selection over positive selection and vice versa for dN/dS >1.0). Based on this metric, we surveyed the degree of functional selections for mitochondrial mutations with different single-cell heteroplasmy levels. For missense mutations, significant negative selections were observed only in homoplasmic mutations. For nonsense mutations, negative selections became noticeable when the single-cell heteroplasmy level exceeded 0.1 (fraction). Notably, the majority of the mutations detected by RedeeM show low heteroplasmy levels (lower than 0.1). These mutations have an dN/dS ratio around 1, suggesting an overall neutrality (**Extended Data Fig. 4a-b**). This is consistent with our understanding that mutant alleles at low heteroplasmy level contribute minimally among predominant wildtype alleles, and therefore, their impact on cellular fitness is likely negligible. We also extended our analysis to a subset of mutations that present in a large percentage of cells (>1%) to examine their potential functional implications on cellular proliferation. Through dN/dS analysis, we did not detect significant selections for either missense or nonsense mutations, which suggests an overall neutrality (**Extended Data Fig. 4c**).

Lastly, we focused on the lineage restricted mitochondrial mutations that are enriched in certain cell types or differentiation trajectories, from which we inferred lineage relationships and cell type origins (**Fig. 2g, Supplementary Fig. 5e**). We examined the potential functional impact for these

mutations. We found that these mutations can appear on all coding genes without significant preference across different cell types or differentiation stages. The dN/dS analysis also suggests no significant selections for these mutations (**Extended Data Fig. 4d-e**).

In summary, these analyses suggest that most mitochondrial mutations utilized by ReDeeM exhibit low heteroplasmy levels and minimal selections and can serve as neutral tags. However, we acknowledge that the functional impact is a very important aspect to consider and report. We have incorporated a functional analysis module into ReDeeM-R to report all these annotations and selection statistics.

Enumeration of multifaceted ReDeeM validations

Both technical benchmarks and biological sanity checks are equally important to support the improved capability of ReDeeM in tracking clones/subclones and inferring genealogies . Here, we enumerated multiple lines of independent evidence, including WGS analysis and numerous other supporting elements that are worthwhile to be highlighted. These includes:

1. Reconstruction of the major known hematopoietic lineages (deciphering genealogies): In **Fig 2 and Extended Data Fig. 8,** we demonstrated RedeeM's capability of deciphering genealogies, where we can use mito mutations to largely reconstruct the expected hematopoiesis differentiation process which requires subclonal level resolution.
2. Detecting clonal expansion (clonal and subclonal tracking): With the expanded cohort, we show that we can detect oligoclonal structure alteration in all older individuals, but none of the younger individuals, which is in strong agreement with our emerging understanding of the high prevalence of clonal hematopoiesis with aging including the very recent work from Mitchell and colleagues[25]. Critically, our work also observes output biases in expanded clones. These findings not only validate the capability of clonal and subclonal tracking, but also beyond what was possible in the single colony WGS studies which lost all information about cell state.
3. Phenotypic recapitulation of ASXL1 mutant CHIP (clonal tracking): The erythroid biases in expanded clones revealed by ReDeeM are consistent with phenotypic measurement in the Asxl1 mutant mouse models[55,56](**Extended Data Fig. 10j)**. This provides further validation at a functional level.
4. The robust HSC-progeny assignment from independent sampling timepoints (clonal tracking and deciphering genealogies): The HSC-progeny assignment between differentiated blood cells, progenitors and HSCs is highly reproducible and reveals sustainable clonal specific state- and behavior biases over time, which not only supports the ability of deciphering genealogies but also provides new and previously unachievable insights into HSC biology (**Fig. 3-4, Supplementary Fig. 7**).
5. Comparison between CRISPR-based lineage tracers versus ReDeeM (deciphering genealogies): This represents the most stringent test of the ability of ReDeeM to report on genealogies that unfolds subclonal evolution over a short timescale of a few months starting from one single cell. We provide validation on two independent mouse batches across 10 tumors. Statistically robust agreement between CRISPR- and ReDeeM-based

tracing is shown, evidenced quantitatively by single-cell level metrics (AOC), and clonal level Adjusted Rand Index (ARI).

6. Comparison between single colony WGS-based lineage tracing inferred by nuclear genome mutations versus mitochondrial mutations (clonal and subclonal tracking): We analyzed WGS data of 42 single colonies from an MPN patient with known JAK2 V617F somatic mutation. We have seen great consistency between confident mitochondrial mutations and the nuclear genome-based tree in both clonal and subclonal level. Of note, we also demonstrate the substantially advanced detectability of true mitochondrial mutations in ReDeeM compared to WGS, especially for lower heteroplasmic variants.

Supplementary references

82. Rausser, S. *et al.* Mitochondrial phenotypes in purified human immune cell subtypes and cell mixtures. *Elife* **10**, (2021).

83. Laricchia, K. M. *et al.* Mitochondrial DNA variation across 56,434 individuals in gnomAD. *Genome Res.* **32**, 569–582 (2022).

84. Thibodeau, A. *et al.* AMULET: a novel read count-based method for effective multiplet detection from single nucleus ATAC-seq data. *Genome Biol.* **22**, (2021).

85. Hao, Y. *et al.* Integrated analysis of multimodal single-cell data. *Cell* **184**, 3573-3587.e29 (2021).

86. Rauluseviciute, I. *et al.* JASPAR 2024: 20th anniversary of the open-access database of transcription factor binding profiles. *Nucleic Acids Res.* (2023) doi:10.1093/nar/gkad1059.

87. Subramanian, A. *et al.* Gene set enrichment analysis: a knowledge-based approach for interpreting genome-wide expression profiles. *Proc. Natl. Acad. Sci. U. S. A.* **102**, 15545–15550 (2005).

88. Heinz, S. *et al.* Simple combinations of lineage-determining transcription factors prime cis-regulatory elements required for macrophage and B cell identities. *Mol. Cell* **38**, 576–589 (2010).

89. Martincorena, I. *et al.* Universal patterns of selection in cancer and somatic tissues. *Cell* **171**, 1029-1041.e21 (2017).
