# Supplementary material for: Deciphering cell states and genealogies of human haematopoiesis
Source: Nature. 2024 Jan 22;627(8003):389–98. doi: 10.1038/s41586-024-07066-z (PMC10937407; doi:10.1038/s41586-024-07066-z)
Supplement: Supplementary file 2 — Reporting Summary [file 41586_2024_7066_MOESM2_ESM.pdf]

nature portfolio

Corresponding author(s): Vijay Sankaran; Jonathan Weissman

Last updated by author(s): Jan 1, 2024

Reporting Summary

Nature Portfolio wishes to improve the reproducibility of the work that we publish. This form provides structure for consistency and transparency in reporting. For further information on Nature Portfolio policies, see our Editorial Policies and the Editorial Policy Checklist.

Statistics

For all statistical analyses, confirm that the following items are present in the figure legend, table legend, main text, or Methods section.

n/a	Confirmed	
☐	☒	The exact sample size (*n*) for each experimental group/condition, given as a discrete number and unit of measurement
☐	☒	A statement on whether measurements were taken from distinct samples or whether the same sample was measured repeatedly
☐	☒	The statistical test(s) used AND whether they are one- or two-sided<br>*Only common tests should be described solely by name; describe more complex techniques in the Methods section.*
☐	☒	A description of all covariates tested
☐	☒	A description of any assumptions or corrections, such as tests of normality and adjustment for multiple comparisons
☐	☒	A full description of the statistical parameters including central tendency (e.g. means) or other basic estimates (e.g. regression coefficient) AND variation (e.g. standard deviation) or associated estimates of uncertainty (e.g. confidence intervals)
☐	☒	For null hypothesis testing, the test statistic (e.g. *F*, *t*, *r*) with confidence intervals, effect sizes, degrees of freedom and *P* value noted<br>*Give P values as exact values whenever suitable.*
☒	☐	For Bayesian analysis, information on the choice of priors and Markov chain Monte Carlo settings
☒	☐	For hierarchical and complex designs, identification of the appropriate level for tests and full reporting of outcomes
☐	☒	Estimates of effect sizes (e.g. Cohen's *d*, Pearson's *r*), indicating how they were calculated

Our web collection on statistics for biologists contains articles on many of the points above.

Software and code

Policy information about availability of computer code

Data collection	For cell sorting and gating, BD FACSDiva software was used
Data analysis	cellranger-arc-2.0.0 was used for single cell RNA+ATAC preprocessing.<br>Amulet v1.1 (https://github.com/UcarLab/AMULET) was used for doublet removal<br>ART-MountRainier-2016-06-05 was used for next-gen sequencing reads simulation<br>Cassiopeia 2.0.0(https://github.com/YosefLab/Cassiopeia) was used for CRISPR lineage tracing data<br>redeemV v1.0.0 (https://github.com/chenweng1991/redeemV) was used for ReDeeM data preprocessing<br>redeemR v1.0.0 (https://github.com/chenweng1991/redeemR) was used for downstream phylogenetic and integrative analysis<br>ScEasyMode 1.0.1 was used for CellHashing demultiplexing<br>Seurat v4.3.0 and Signac v1.5.0 was used for single cell multimodal analysis<br>SCAVENGE(https://github.com/sankaranlab/SCAVENGE) was used for HSC progeny analysis<br>jungle(https://github.com/felixhorns/jungle) was used for phylogenetic fitness analysis<br>FIMO from meme-5.4.1 was used for TF motif scanning

For manuscripts utilizing custom algorithms or software that are central to the research but not yet described in published literature, software must be made available to editors and reviewers. We strongly encourage code deposition in a community repository (e.g. GitHub). See the Nature Portfolio guidelines for submitting code & software for further information.

Data

Policy information about availability of data

All manuscripts must include a data availability statement. This statement should provide the following information, where applicable:

- Accession codes, unique identifiers, or web links for publicly available datasets
- A description of any restrictions on data availability
- For clinical datasets or third party data, please ensure that the statement adheres to our policy

All data generated in the manuscript have been deposited in GEO (GSE219015). The processed Seurat objects are available on figshare: https://doi.org/10.6084/m9.figshare.23290004. The processed mutation calling files on figshare: https://doi.org/10.6084/m9.figshare.24418966.v1. Single colony WGS data are from dbGAP:phs002308.v1.p1. TF motif database JASPAR2020 (https://jaspar2020.genereg.net/) was used with ChromVar. HOCOMOCOv11 (https://hocomoco11.autosome.org/downloads_v11) Human TF database was used for FIMO analysis.

Human research participants

Policy information about studies involving human research participants and Sex and Gender in Research.

Reporting on sex and gender	We have used the term sex or gender carefully throughout the manuscript. Findings are applied generally. Genders were considered in study design. Gender was determined based on self-report. Informed consent was obtained under a sample banking protocol that was approved by the IRB of Mass General Brigham and Boston Children's Hospital
Population characteristics	Age is an important characteristic. We have collected 7 young (range from 20 to 32 yo) and 4 aged donors (range from 69 to 85 yo) and performed systematic comparison across age groups
Recruitment	Participants were recruited with self report. Donors with reported conditions or diseases were excluded, but specific screens were performed. These donors were considered as in general healthy donors
Ethics oversight	The Fresh bone marrow samples from healthy young donors were aspirated with informed consent under a sample banking protocol that was approved by Institutional Review Board (IRB) of Boston Children's Hospital. The sternum bone marrow from aged donors was collected following sternotomy for cardiac surgery with informed consent under a sample banking protocol that was approved by the IRB of Mass General Brigham.

Note that full information on the approval of the study protocol must also be provided in the manuscript.

Field-specific reporting

Please select the one below that is the best fit for your research. If you are not sure, read the appropriate sections before making your selection.

☒ Life sciences ☐ Behavioural & social sciences ☐ Ecological, evolutionary & environmental sciences

For a reference copy of the document with all sections, see nature.com/documents/nr-reporting-summary-flat.pdf

Life sciences study design

All studies must disclose on these points even when the disclosure is negative.

Sample size	No sample size calculations were performed a priori. Analyses involved thousands of cells per comparison, providing a robust sample size in-line with similar high-throughput scRNA-seq comparisons and technologies. To well cover different hematopoietic cell populations and clones. We sampled large sample sizes for main donors: 54,221 cells for young-1, 34,721 cells for young-2; 20,496 cells for aged-1; 25,717 dells for aged-2
Data exclusions	The data preprocessing for the joint single-cell RNA and ATAC data was performed using 10X Genomics data preprocessing software Cell-ranger-arc. The basic quality control analysis was as follows: RNA UMI: 1,000~25,000 transcripts per cell, unique ATAC fragment: 1,000 ~ 70,000, Fragment on peak minimum percentage: 10%, minimum mtDNA copies per position per cell: 10X. Finally, the possible doublets are removed by Amulet (using default parameter).
Replication	All findings discussed in the manuscript are reproducible in two independent sampling from the same individuals as well as two independent individuals.
Randomization	There were no variables or interventions to randomize in this study.
Blinding	Blinding is not relevant to our study. Analyses were performed in an exploratory manner where blinding is not possible.

Reporting for specific materials, systems and methods

We require information from authors about some types of materials, experimental systems and methods used in many studies. Here, indicate whether each material, system or method listed is relevant to your study. If you are not sure if a list item applies to your research, read the appropriate section before selecting a response.

Materials & experimental systems

n/a	Involved in the study	
☐	☒	Antibodies
☐	☒	Eukaryotic cell lines
☒	☐	Palaeontology and archaeology
☒	☐	Animals and other organisms
☒	☐	Clinical data
☒	☐	Dual use research of concern

Methods

n/a	Involved in the study	
☒	☐	ChIP-seq
☒	☐	Flow cytometry
☒	☐	MRI-based neuroimaging

Antibodies

Antibodies used

To further enrich the hematopoietic stem cells (HSCs), an aliquot of the enriched CD34+ cells were stained by one of the following two antibody panels. 1) CD34 PerCP-Cy5.5 (Catalog #347222); CD45RA Alexa Fluor 488 (Catalog #304114); CD90 PE-Cy7 (Catalog #561558); and DAPI (Catalog #D1306) as viability dye. Or 2) CD34 BV421 (Catalog #562577); CD45RA-APC-H7 (Catalog #560674); CD90 PE-Cy7 (Catalog #561558), and 7-AAD as viability dye(Catalog #559925). The cells were further sorted using BD FACSAriaTM for CD34+CD45RA-CD90+ to enrich HSCs. All antibodies are from BD biosciences.

Validation

Each lot of these antibodies is quality control tested by immunofluorescent staining with flow cytometric analysis. The validations are routinely done in the Sankaran lab by staining human hematopoietic stem and progenitor cells.

Eukaryotic cell lines

Policy information about cell lines and Sex and Gender in Research

Cell line source(s)

K-562 cell line from ATCC

Authentication

None of the cell line was authenticated

Mycoplasma contamination

Cell lines are routinely tested for mycoplasma contamination. Results were consistently negative.

Commonly misidentified lines
(See ICLAC register)

No commonly misidentified lines were used as part of this study.
