# Supplementary material for: Deciphering cell states and genealogies of human haematopoiesis
Source: Nature. 2024 Jan 22;627(8003):389–98. doi: 10.1038/s41586-024-07066-z (PMC10937407; doi:10.1038/s41586-024-07066-z)
Supplement: Supplementary file 3 — This file contains the ReDeeM protocol. [file 41586_2024_7066_MOESM3_ESM.pdf]

ReDeeM protocol(2023-11-20) Sankaran & Weissman lab

Introduction

Protocol for single-cell **Re**gulatory multi-omics with **Dee**p **M**itochondrial mutation profiling.

Following this protocol, three separate libraries plus one optional library (mtDNA lib; RNA lib, ATAC lib, and an optional Hashing library) will be generated in parallel for joint single-cell profiling.

This protocol has been tested for hematopoietic stem/progenitor and differentiated cells (Including T cells, B cells, NK cells, monocyte, dendritic cells, plasma, GMP, MDP, CLP, MPP, HSC, etc). For other tissues or cell types, some conditions may need to be further optimized.

by Chen Weng (cweng@wi.mit.edu, cweng@broadinstitute.org)
Michael Poeschla (mpoeschl@broadinstitute.org),
Alessandro Hammond (ahammond@college.harvard.edu)

Materials

- (Optional) TotalSeq™-A0301 anti-mouse Hashtag Antibody (A0301-A0310)
- (Optional) TruStain FcX™ (BioLegend, # 422302)
- Pierce™ 16% Formaldehyde (w/v), Methanol-free (Thermo Scientific, # 28906)
- BSA (Sigma, A4503-100G)
- Nonidet-P40 substitute (USbiological CAS: 9036-19-5)
- 10X Genomics multiome kit (10X Genomics, PN-1000283/PN-1000285)
- glyco-diosgenin, GDN (Avanti Polar Lipids, 850525P-500MG)
- NEBNext HF PCR MM (NEB, # M0541L)
- Protector RNase Inhibitor (Sigma, # 3335402001)
- xGen™ Hybridization and Wash v2 Reagents, 16 rxn (IDT, # 10010351)
- xGen™ Hybridization and Wash v2 Beads, 16 rxn (IDT, # 10010353)
- xGen™ Universal Blockers NXT (IDT, # 1079584)
- KAPA HiFi HotStart ReadyMix (Kapa Biosystems, KK2601)
- SYBR Green I Nucleic Acid Gel Stain - 10,000X concentrate in DMSO (Life Technologies # S7563)
- Agilent High Sensitivity DNA Kit (Agilent # 5067-4626)
- In-house buffers
  - Fixation buffer： 0.1% Formaldehyde， 0.04% BSA， 0.2U/ul RNase inhibitor in DPBS
  - FACS buffer: 2% BSA in DPBS
  - DPBS/RI buffer: 0.04% BSA, 0.2U/ul RNase Inhibitor in DPBS
  - Perm buffer: 10mM Tris-HCl, 10mM NaCl, 3mM MgCl2, 0.1% NP40, 1mM DTT, 1 U/ul Rnase inhibitor in ddH2O

- Perm-Wash buffer: 10mM Tris-HCl, 10mM NaCl, 3mM MgCl2, 0.02% BSA, 1mM DTT, 0.2 U/ul RNase inhibitor in ddH2O
- Resuspension buffer: 1mM DTT and 1U/ul Rnase inhibitor in 1X nuclei buffer from multiome kit (10X Genomics, PN-1000283/PN-1000285)
- ATAC-GDN buffer: 1% GDN in 1X ATAC buffer (PN-2000193)

Oligo sequences

- (Optional) HTO additive primer： 5'-GTGACTGGAGTTCAGACGTGTGCTCTTCCGAT*C*T-3'
- (Optional) D701_LONG primer: 5'-CAAGCAGAAGACGGCATACGAGATCGAGTAATGTGACTGGAGTTCAGACGTGTGCTCTTCCGAT*C*T-3'
- (Optional) Hash-primer: 5'-AATGATACGGCGACCACCGAGATCTACACTCTTTCCCTACACGACGC*T*C-3'
- Nextera oligos: N701-N712: 5'-CAAGCAGAAGACGGCATACGAGAT[NNNNNNNN]GTCTCGTGGGCTCGG-3'
- P5: 5'-AATGATACGGCGACCACCGAGA-3'
- P7: 5'-CAAGCAGAAGACGGCATACGAGAT-3'
- Mitochondrial DNA capture probes: Probe sequences will be available soon (4 sets of staggered probes mitoV1, mitoV2, mitoV3, mitoV4 with 137 probes (100nt) in each probe set).

Procedure

(Optional) Cell Hashing for multiple samples

1. Centrifuge 100K ~ 1M Bone Marrow Mononuclear Cells (BMMCs) for each sample at 500g for 5 min.
2. Resuspend in 50 µl FACS buffer.
3. Add 5 µl Human TruStain FcX™ in each sample, mix and incubate at 4°C for 10 minutes to for blocking.
4. During the incubation, for each sample, dilute 1 µl TotalSeqTM antibody (unique for each sample) in 50 µl FACS buffer in a corresponding fresh, 1.5 ml, low-binding tube
5. Transfer the corresponding 50 ul antibody dilution from step 4 into each of the blocked-cell suspensions, and incubate at 4°C for 30 minutes.
6. Add 3 ml FACS buffer in each sample and centrifuge 500g at 4°C for 5 minutes for washing.
7. Remove supernatant and repeat the wash 1 more time with 3ml FACS buffer for each sample and centrifuge 500g 4°C for 5 min
8. Pool Hashed samples together (usually equal cell number) as one sample to proceed forward.

Fixation and permeabilization

We found that decreasing BSA concentration in most of the buffers can improve the mtDNA recovery rate. The in-house buffers provided in Material section are optimized for hematopoietic cells. The recipes can be further optimized for different tissues and cell types.

9. For each sample, resuspend cells in 1ml **Fixation buffer**, and incubate at room temperature for 10 minutes.

10. Quench the fixation reaction with 50 ul glycine (2.5M) at final concentration of 0.125M, and incubate at room temperature for 3 minutes.

11. Centrifuge 500g at 4°C for 5 minutes.

12. Remove supernatant, add 3ml cold **PBS/RI**, and centrifuge 500g at 4°C for 5 minutes.

13. Remove supernatant, add 1ml **Perm buffer,** and incubate for 5 minutes on ice. Then immediately add **Perm-Wash buffer** 3ml, and centrifuge 500g at 4°C for 5 minutes.

14. Remove supernatant, add 200ul **Resuspension buffer,** and centrifuge 500g at 4°C for 5 minutes.

15. Remove the top of supernatant without disturbing cell pellet, leaving 20~100 ul **Resuspension buffer,** and resuspend and count the cells. Ideally, make the cell concentration equal to or more than 2800 cell/ul

Tagmentation with GDN

> *We found that adding GDN during tagmentation can increase the mtDNA recovery rate without significantly affecting RNA and ATAC quality. But standard tagmentation reaction without GDN also works to proceed forward.*

16. Generate the following mix per sample for tagmentation:

Table1

	A	B
1	Name	Volume per reaction (ul)
2	ATAC buffer B (10x Genomics, PN-2000193)	5.5
3	ATAC-GDN buffer (In-house buffer)	1.5
4	ATAC Enzyme B (10x Genomics, PN-2000265/2000272)	3
5	Cells at a concentration of 2800 cells/µl (Step 15)	5

17. Incubate each sample at 37°C for 1 hour, and immediately proceed to next step.

GEM Generation & Barcoding

18. Use the tagmented cells from step 17 to proceed, following [10x Genomics multiome user guide revB CG000338-Step2] for GEM generation and barcoding

Post GEM Incubation Cleanup

19. Following [10x Genomics multiome user guide revB CG000338-Step3] for Post GEM Incubation Cleanup.

Pre-Amplification PCR

20. Follow [10x Genomics multiome user guide revB CG000338-Step4], except

a) (Optional, if cell hashing was performed) Spike in 1 ul 0.2uM **HTO additive primer** at [10x Genomics multiome user guide revB CG000338-Step4.1]

b) Elute in 100ul Buffer EB (CG000338-Step4.3) to generate Pre-Amp library

ATAC library prep (Small-scale pilot test)

21. Use 1 µL of Pre-Amp library in pilot test for ATAC PCR with monitoring using SYBR Green

Table2

	A	B
1	Component	Volume per reaction (ul)
2	Pre-Amp (Step 20)	1
3	NEBnext mix	12.5
4	N701 primer	1.2
5	SI PCR primer B(10X Genomics, PN-2000128)	1.2
6	100X SYBR green	0.25
7	ddH2O	8.85
8	Total	25

Run qPCR using the following program: 1. 98 °C 45s; 2. 98 °C 10s; 3. 63 °C 30s; 4. 72 °C 20s; 5. Read Plate; 6. Go to step 2 for 35 more cycles. Stop the qPCR manually based on the amplification curve. Usually around 10 or below 10 cycles when the curve is reaching plateau.

22. Run Bioanalyzer for the qPCR product using Agilent High Sensitivity DNA Kit. The Expected ATAC traces are as below. Different cell types may show slightly different trace.

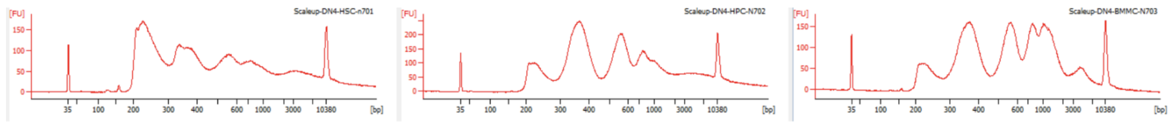

ATAC library prep (Scale-up)

23. Scale up the PCR above to 8 wells per sample, (50 µL per well, for a total 400 µL PCR mix), monitored using SYBR Green. Each well should contain::

Table3

	A	B
1	Component	Volume per reaction (ul)
2	Pre-Amp	5
3	NEBnext mix	25
4	N701	2.4
5	SI PCR primer B(10X Genomics, PN-2000128)	2.4
6	100X SYBR Green	0.5
7	ddH2O	14.7
8	Total	50

Run qPCR using the same program in small-scale test: 1. 98 °C 45s; 2. 98 °C 10s; 3. 63 °C 30s; 4. 72 °C ***60s;*** 5. Read Plate; 6. Go to step 2 for 35 more cycles. Stop the qPCR manually based on the amplification curve. **Limit the number of cycles to 10 or lower.**

24. Purify the PCR product.

    Combine each 2 wells (into 100ul) for purification. Purify by SPRI beads with size selection 0.4X--1.55X. Elute in 20ul*4 ddH2O (80ul total), The expected DNA yield is more than 3000 ng.

25. Run Bioanalyzer for the qPCR product using Agilent High Sensitivity DNA Kit.

26. Ready for sequencing. We recommend paired-end illumina sequencing. R1: 150nt, i7: 8nt, i5: 24nt, R2:150nt. 20K-25K sequencing reads are suggested.

27. The product from step 25 is also ready for mitochondrial DNA capture. Typically, 2000 ng is needed.

Mitochondrial DNA (mtDNA) library prep

28. For each sample, prepare 500ng ATAC library X4 (2000ng in total) for four hybridization reactions using staggered probes (mitoV1, mitoV2, mitoV3, mitoV4)

29. The hybridization is performed by following IDT xGen™ hybridization capture guide. The updating protocol can be found on IDT website. Briefly, the capture is a two-day protocol as shown below.

30. D1: Setting up hybridization reaction following APPENDIX A in xGen™ hybridization capture guide. xGen Hyb Panel is replaced by one of the probes for each reaction: mitoV1/mitoV2/mitoV3/mitoV4. The hybridization reaction is as below:

Table4

	A	B
1	Component	Volume per reaction (ul)
2	xGen 2X Hybridization Buffer	9.5
3	xGen Hybridization Buffer Enhancer	3
4	xGen Universal Blocker NXT	2
5	Probe mitoV1/mitoV2/mitoV3/mitoV4	4.5
6	Total	19

The hybridization reaction is performed 95 °C 30s, followed by 65 °C 16h

16:00:00

31. D2: Capture and washes are performed following Tube protocol xGen™ hybridization capture guide (from Page 23). The updating protocol can be found on IDT website

32. The post-capture PCR is monitored by SYBR: 1 well of PCR (as below) is performed for each hybridization reaction.

Table5

	A	B
1	Component	Volume per reaction (ul)
2	Beads after washes	19.7
3	KAPA ReadyMix	25
4	P5 primer	2.4
5	P7 primer	2.4
6	SYBR	0.5
7	Total	50

Run qPCR using the following program: 1. 98 °C 45s; 2. 98 °C 10s; 3. 63 °C 30s; 4. 72 °C 20s; 5. Read Plate; 6. Go to step 2 for 35 more cycles. Stop the qPCR manually based on the amplification curve. **Limit the number of cycles to 3-5 cycles.**

33. Combine every four wells for each sample (corresponding to 4 hybridizations using probes mitoV1, mitoV2, mitoV3, mitoV4) in total 200ul and purify by 1.6X SPRI beads (320 ul SPRI each), finally elute in 25 ul ddH2O.

34. Run Bioanalyzer for the purified PCR product using Agilent High Sensitivity DNA Kit. The expected mtDNA fragment trace look like below:

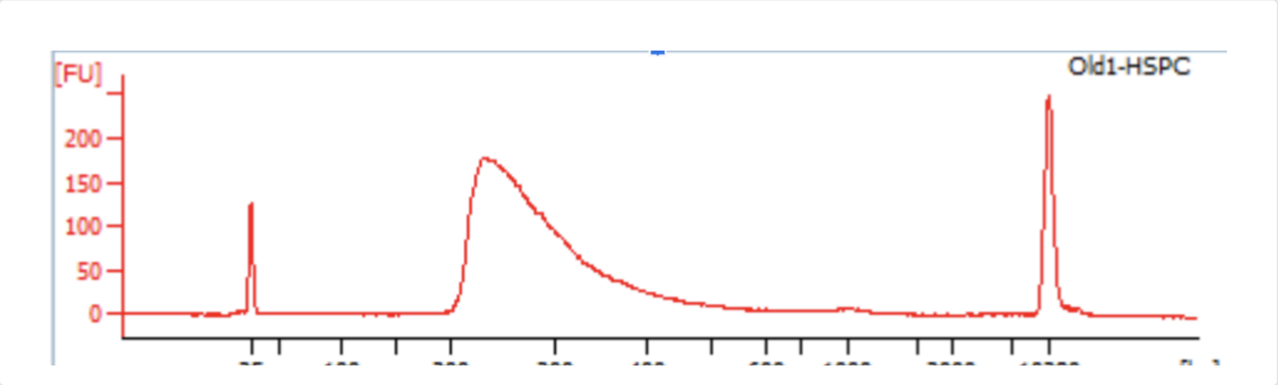

35. Ready for sequencing. For sensitive variant calling, we recommend paired-end illumina sequencing. R1: 150nt, i7: 8nt, i5: 24nt, R2:150nt. We recommend deep sequencing with 50K-60K sequencing reads per cell for mtDNA library.

RNA library

36. RNA library is prepared following [10x Genomics multiome user guide revB CG000338-Step6 and Step7]

37. Ready for sequencing. We recommend paired-end illumina sequencing following 10x Genomics guide: R1: 28nt, i7: 10nt, i5: 10nt, R2:90nt.

(Optional) Hashing library

38. If cell hashing was performed, the hashing library is prepared using PCR as below, which is also monitored by SYBR

Table6

	A	B
1	Component	Volume per reaction (ul)
2	Pre-Amp	5
3	NEBnext mix	12.5
4	D701_LONG	1.2
5	Hash-primer	1.2
6	100X SYBR Green	0.25
7	ddH2O	4.85
8	Total	25

Run hashing qPCR using the following program: 1. 98 °C 45s; 2. 98 °C 10s; 3. 63 °C 30s; 4. 72 °C 20s; 5. Read Plate; 6. Go to step 2 for 35 more cycles. Stop the qPCR manually based on the amplification curve. Usually 15~20 cycles are needed.

Data analysis pipeline

39. ReDeeM-V for data preprocessing (Command line tools)

40. ReDeeM-R for downstreme analysis (R package)
